# 12-h clock regulation of genetic information flow by XBP1s

**DOI:** 10.1371/journal.pbio.3000580

**Published:** 2020-01-14

**Authors:** Yinghong Pan, Heather Ballance, Huan Meng, Naomi Gonzalez, Sam-Moon Kim, Leymaan Abdurehman, Brian York, Xi Chen, Yisrael Schnytzer, Oren Levy, Clifford C. Dacso, Colleen A. McClung, Bert W. O’Malley, Silvia Liu, Bokai Zhu

**Affiliations:** 1 UPMC Genome Center, Pittsburgh, Pennsylvania, United States of America; 2 Aging Institute of UPMC, University of Pittsburgh School of Medicine, Pittsburgh, Pennsylvania, United States of America; 3 Department of Molecular and Cellular Biology, Baylor College of Medicine, Houston, Texas, United States of America; 4 Translational Neuroscience Program, Department of Psychiatry, Center for Neuroscience, University of Pittsburgh, Pittsburgh, Pennsylvania, United States of America; 5 The Mina & Everard Goodman Faculty of Life Sciences, Bar-Ilan University, Ramat-Gan, Israel; 6 Department of Pathology, University of Pittsburgh School of Medicine, Pittsburgh, Pennsylvania, United States of America; 7 Pittsburgh Liver Research Center, University of Pittsburgh, Pennsylvania, United States of America; 8 Division of Endocrinology and Metabolism, Department of Medicine, University of Pittsburgh School of Medicine, Pittsburgh, Pennsylvania, United States of America; Charité - Universitätsmedizin Berlin, GERMANY

## Abstract

Our group recently characterized a cell-autonomous mammalian 12-h clock independent from the circadian clock, but its function and mechanism of regulation remain poorly understood. Here, we show that in mouse liver, transcriptional regulation significantly contributes to the establishment of 12-h rhythms of mRNA expression in a manner dependent on Spliced Form of X-box Binding Protein 1 (XBP1s). Mechanistically, the motif stringency of XBP1s promoter binding sites dictates XBP1s’s ability to drive 12-h rhythms of nascent mRNA transcription at dawn and dusk, which are enriched for basal transcription regulation, mRNA processing and export, ribosome biogenesis, translation initiation, and protein processing/sorting in the Endoplasmic Reticulum (ER)-Golgi in a temporal order consistent with the progressive molecular processing sequence described by the central dogma information flow (CEDIF). We further identified GA-binding proteins (GABPs) as putative novel transcriptional regulators driving 12-h rhythms of gene expression with more diverse phases. These 12-h rhythms of gene expression are cell autonomous and evolutionarily conserved in marine animals possessing a circatidal clock. Our results demonstrate an evolutionarily conserved, intricate network of transcriptional control of the mammalian 12-h clock that mediates diverse biological pathways. We speculate that the 12-h clock is coopted to accommodate elevated gene expression and processing in mammals at the two rush hours, with the particular genes processed at each rush hour regulated by the circadian and/or tissue-specific pathways.

## Abstract 2

Distinct from the well-known 24-hour circadian clock, this study shows that the mammalian 12-hour clock upregulates genetic information flow capacity during the two "rush hours" (dawn and dusk) in a manner dependent on the transcription factor XBP1s.

## Introduction

All life on earth is governed by biological rhythms that are defined as self-sustained oscillations cycling with a fixed period. Biological clocks enable organisms to keep track of the time of day and to adjust their physiology to recurring daily changes in the external environment, including nutrient and microenvironment status. Our understandings of biological rhythms in mammals have expanded beyond the well-characterized circadian rhythms (approximately 24-h oscillation) in recent years through the discovery of the existence of 12-h rhythms in mice [1,2]. A handful of studies followed up on these initial studies and have proposed different hypotheses regarding how 12-h rhythms are established [1,3–5]. Early studies favor the hypothesis that the mammalian 12-h rhythms are not cell autonomous and instead are established by the combined effects of circadian clock and fasting-feeding cues. This conclusion was largely based upon the findings showing the lack of cell-autonomous 12-h rhythms of gene expression in forskolin-synchronized NIH3T3 cells and altered 12-h rhythms of gene expression under certain feeding and circadian clock perturbation conditions [2–4]. Alternatively, it was suggested that 2 circadian transcription activators or repressors appearing in antiphase are theoretically capable of establishing 12-h rhythms of gene expression in a cell-autonomous manner [5]. Contrary to these hypotheses, our group discovered that the mammalian 12-h rhythms are not only cell autonomous, but they are also established by a dedicated “12-h clock” separate from the circadian clock and function to coordinate cellular stress with metabolism [1,6,7].

The main line of evidences supporting the existence of a cell-autonomous mammalian 12-h clock include (1) the presence of intact hepatic 12-h rhythms of gene expression in circadian-clock–deficient mice in vivo under free-running conditions [1,6]; (2) the detection of cell-autonomous 12-h rhythms of gene expression in mouse embryonic fibroblasts (MEFs) in a *Bmal1*-independent manner [1,6]; (3) that similar genes are regulated in a 12-h rhythmic manner in different organisms, indicating evolutionary conservation of these 12-h mechanisms [1]; and (4) that genes exhibiting 12-h rhythms arose much earlier during evolution than circadian genes [1,6,8]. It is hypothesized that circatidal clock mechanisms would have developed before the divergence of the major animal clades, existing in a common ancestor, occupying bodies of water in which tidal cycles would have been as ecologically important—if not more so—than the circadian cycle [8–10].

Due to the strong enrichment of unfolded protein response (UPR) pathways in hepatic 12-h transcriptomes, we hypothesized that the mammalian 12-h clock may be regulated transcriptionally by the UPR transcription factor Spliced Form of X-box Binding Protein 1 (XBP1s) [6]. In agreement with this hypothesis, we previously found that small interfering RNA (siRNA)-mediated knockdown of *Xbp1* in MEFs impaired cell-autonomous 12-h mRNA oscillations of several genes, including *Eif2ak3* and *Sec23b* [1,6]. While these data suggest a role of XBP1s in regulating 12-h rhythms of gene expression in vitro, it remains undetermined whether XBP1s is a major transcriptional regulator of the mammalian hepatic 12-h clock in vivo. In the current study, we addressed this question and discovered that XBP1s contributes significantly to the establishment and maintenance of 12-h rhythms, but not circadian rhythms, at the transcriptional level. Our study therefore demonstrates an intricate network of transcriptional control of the mammalian 12-h clock that mediates diverse biological pathways, including transcription, translation, ribosome biogenesis, mRNA and protein processing, and vesicle trafficking.

## Results

### Liver-specific deletion of XBP1 does not affect the core circadian clock in mice

**Fig 1:**
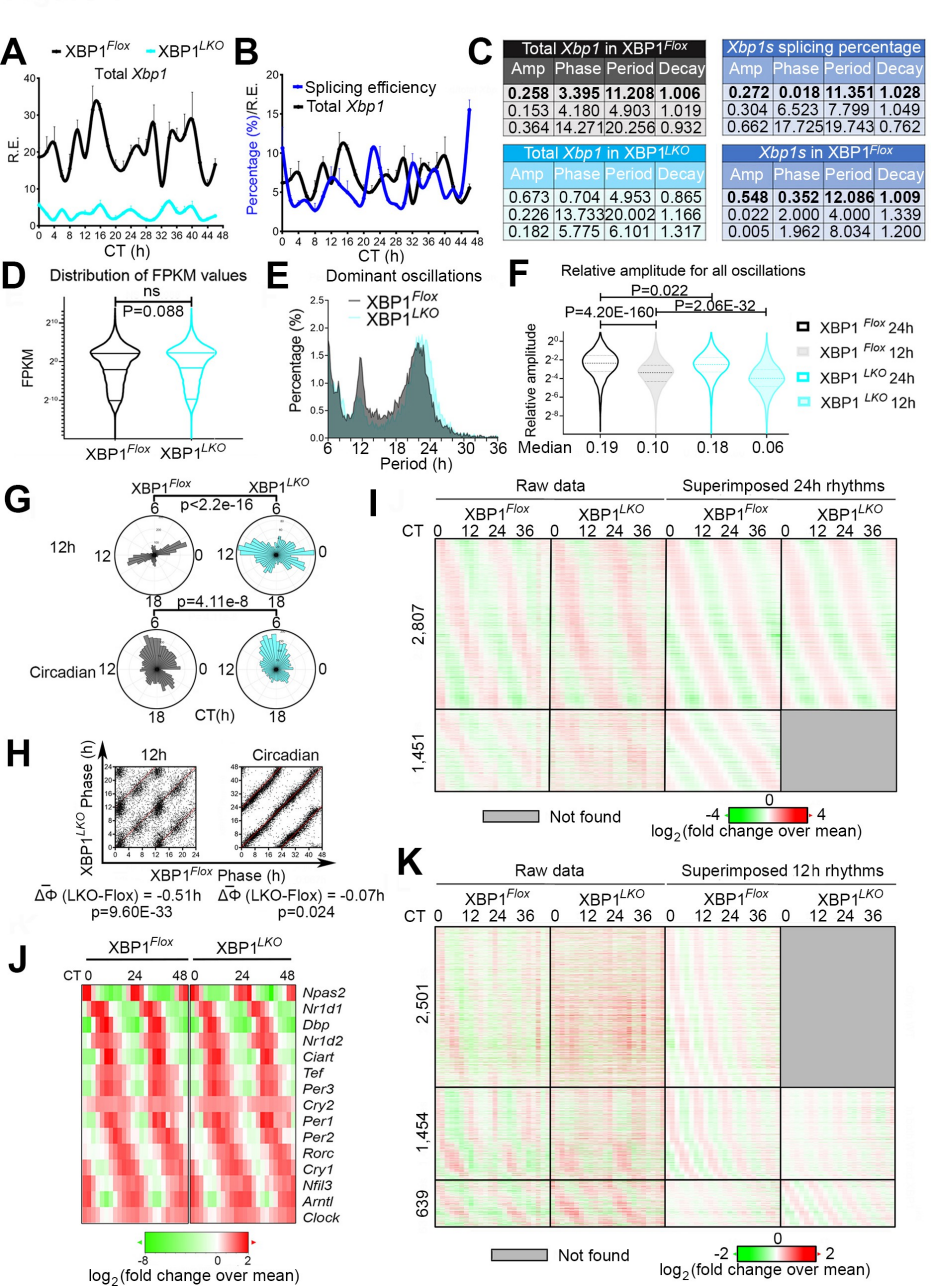
Liver-specific deletion of XBP1s impairs global hepatic 12-h transcriptome but not the circadian rhythm in mice. (A) qPCR analysis of total hepatic *Xbp1* mRNA in wild-type (XBP1^*Flox*^) and XBP1 liver-specific knockout (XBP1^*LKO*^) mice. (B) Calculated splicing efficiency (ratio of *Xbp1s* to total *Xbp1* mRNA) and total hepatic *Xbp1* mRNA in XBP1^*Flox*^ mice. (C) Eigenvalue/pencil analysis of total *Xbp1* mRNA level in XBP1^*Flox*^ and XBP1^*LKO*^ mice, *Xbp1s* mRNA level and the splicing efficiency in XBP1^*Flox*^ mice. (D) Distributions of mean FPKM values in XBP1^*Flox*^ and XBP1^*LKO*^ mice. (E) Distribution of the periods of dominant oscillations identified by the eigenvalue/pencil method from XBP1^*Flox*^ and XBP1^*LKO*^ mice. (F) Distribution and median values of the relative amplitudes of all 12-h and circadian rhythms in XBP1^*Flox*^ and XBP1^*LKO*^ mice. (G) Polar histograms demonstrating the phase distributions of all dominant circadian and 12-h rhythms from XBP1^*Flox*^ and XBP1^*LKO*^ mice. (H) Scatter plot showing the phases of each commonly shared 12-h (left) and circadian (right) genes in XBP1^*Flox*^ and XBP1^*LKO*^ mice. (I) Heat map of all circadian gene expression (or lack thereof) in XBP1^*Flox*^ and XBP1^*LKO*^ mice with both raw data and superimposed 24-h rhythms shown. (J) Heat map of 15 core circadian clock gene expression in XBP1^*Flox*^ and XBP1^*LKO*^ mice with raw data shown. (K) Heat map of all approximately 12-h gene expression (or lack thereof) in XBP1^*Flox*^ and XBP1^*LKO*^ mice with both raw data and superimposed 12-h rhythms shown. Data are graphed as the mean ± SEM (*n* = 2 to 5) for (A) and (B). Numerical values are available in S1 and S2 Data. ΔΦ, phase difference; Amp, amplitude; CT, constant time; FPKM, Fragments Per Kilobase of transcript per Million mapped reads; NS, not significant; qPCR, quantitative PCR; R.E., relative expression; XBP1, X-box binding protein 1.

To delete XBP1 specifically in the liver, we crossed XBP1^*Flox*^ mice (XBP1^*fl/fl*^ mice with loxP sites flanking exon 2 of the *Xbp1* gene [S1A Fig]) [11] with Albumin-CRE transgenic mice as previously described [12]. Liver-specific deletion of XBP1s was confirmed by both quantitative PCR (qPCR) and western blot analysis (Fig 1A–1C, S1B and S1C Fig). Consistent with previous reports [3,6], robust 12-h rhythms of total *Xbp1* as well as spliced form of *Xbp1* (*Xbp1s*) mRNA levels were observed in control (XBP1^*Flox*^) mice but not in XBP1 liver-specific knockout (XBP1^*LKO*^) mice, indicating that XBP1s autoregulates its own 12-h rhythm of expression (Fig 1A–1C). We further observed an approximately 3-h phase delay between the acrophases of *Xbp1s* (0.3 h) and total *Xbp1* (3.4 h) (Fig 1C).

To rule out the potential effects of liver-specific ablation of XBP1s on mouse locomotor activity and/or feeding behavior, which could confound the interpretation of the transcriptome data, we subjected both XBP1^*Flox*^ and XBP1^*LKO*^ mice to home cage and Comprehensive Lab Animal Monitoring System (CLAMS), respectively. As shown in S1D–S1H Fig, liver-specific deletion of XBP1 does not alter the rhythmic locomotor activity or fasting-feeding cycles in mice.

To identify the XBP1s-dependent oscillating transcriptome, we performed RNA sequencing (RNA-Seq) analysis in the liver of XBP1^*Flox*^ and XBP1^*LKO*^ mice at 2-h intervals for a total of 48 h under constant darkness in duplicates (S1 and S2 Tables and see Materials and methods for details). The absence of reads mapped to exon 2 of the *Xbp1* gene in XBP1^*LKO*^ mice further confirmed the successful knockout of the *Xbp1* gene (S1A Fig). Averaged gene expression across 48 h was comparable between XBP1^*Flox*^ and XBP1^*LKO*^ mice (Fig 1D). All superimposed oscillations in either XBP1^*Flox*^ or XBP1^*LKO*^ mice were identified by the newly developed eigenvalue/pencil method [6,7], which (unlike canonical oscillation-identification methods such as JTK_CYCLE and ARSER) does not require a pre-assignment of period range and therefore allows unbiased identification of all superimposed oscillations [6,7]. Consistent with past findings [6], the vast majority of oscillations identified were circadian rhythms and oscillations that cycle at the second (approximately 12 h) and third (approximately 8 h) harmonics of the circadian rhythm (due to the 2-h sampling frequency of the current study, only up to the third harmonics can be identified with high confidence [7]) (Fig 1E and S3 Table). To determine the false discovery rate (FDR) of the identified rhythmic transcripts, we used a permutation-based method that randomly shuffles the time label of gene expression data and subjects all of the permutation dataset to the eigenvalue/pencil method as previously described [13]. As expected, permutation datasets are devoid of distinct populations of oscillations cycling at different harmonics of the circadian rhythm (S2A Fig). In this way, we identified a total of 4,258 circadian genes (21.70 ± 2.58 h) (among which 3,251 have dominant circadian rhythms) with an FDR of 0.07, a total of 4,594 approximately 12-h genes (11.62 ± 0.48 h) (among which 1,709 have dominant 12-h rhythms) with an FDR of 0.21, and a total of 3,792 approximately 8-h cycling genes (among which 910 have dominant approximately 8-h rhythms) with an FDR of 0.31, respectively, in XBP1^*Flox*^ mice (S2B and S2C Fig) (see Materials and methods for details). Agreeing with past findings [6], the average amplitude of 12-h genes are smaller compared to that of circadian genes in XBP1^*Flox*^ mice (Fig 1F). Furthermore, while the phases of circadian rhythms are evenly distributed throughout the day in XBP1^*Flox*^ mice (Fig 1G and 1H), the phases of 12-h rhythms are more enriched at dawn (constant time [CT]0–CT2) and dusk (CT12–CT14), which is more evident for dominant oscillations (Fig 1G).

Of all circadian genes identified in XBP1^*Flox*^ mice, approximately two-thirds (2,807 out of 4,258) were unaffected by hepatic XBP1s ablation, which includes all known core circadian clock genes (Fig 1I and 1J and S2B Fig). On average, comparable average gene expression and phase distribution of circadian oscillations are found between XBP1^*Flox*^ and XBP1^*LKO*^ mice (Fig 1G–1J), although a very small average phase advance of 4 min was observed in XBP1^*LKO*^ mice that reached statistical significance (*P* = 0.024) (Fig 1H). We believe this phase difference most likely resulted from technical bias because we always euthanized XBP1^*Flox*^ mice first and therefore the actual circadian time should be a few minutes later than what is reported for the XBP1^*LKO*^ mice. Circadian genes not affected by hepatic XBP1s ablation are enriched in circadian rhythm and metabolic pathways (S2D Fig), which are the dominant biological pathways under hepatic circadian clock control [14,15]. We further identified 1,451 genes, whose superimposed circadian rhythms are not found in XBP1^*LKO*^ mice according to the eigenvalue/pencil method (Fig 1I). The top enriched Gene Ontology (GO) terms include a number of metabolic pathways (S2E Fig). A closer examination revealed that many of these genes have a period just falling out of the 21-h to 25-h range in XBP1^*LKO*^ mice and were thus deemed not to have circadian rhythm (such as the *Ppox* gene shown in S2E Fig). We conjecture that the circadian rhythms of these genes are likely not driven by the circadian clock and are altered as an indirect consequence of XBP1s ablation, with a possibility that these circadian rhythms are impaired at the post-transcriptional level in XBP1^*LKO*^ mice. Taken together, our data indicate that the liver-specific deletion of XBP1 does not affect the core circadian clock in mice.

### Liver-specific deletion of XBP1 globally impairs the 12-h transcriptome, which is enriched in pathways regulating central dogma information flow

**Fig 2:**
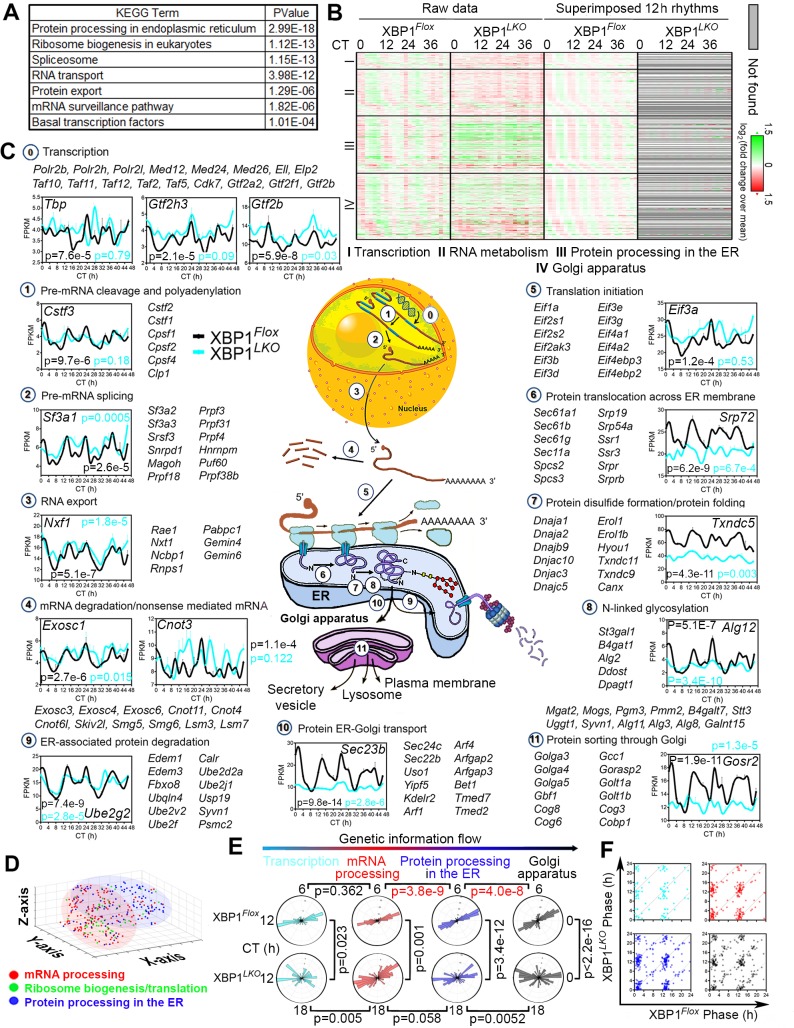
XBP1s-dependent hepatic 12-h transcriptome is enriched in regulating CEDIF. (A) GO analysis showing top-enriched KEGG pathways and their corresponding *P* values for XBP1s-dependent 12-h transcriptome. (B) Heat map of 12-h cycling gene expression (or lack thereof) involved in transcription (I), mRNA metabolism (II), protein metabolism in the ER (III) and the Golgi apparatus (IV) in XBP1^*Flox*^ and XBP1^*LKO*^ mice with both raw data and superimposed 12-h rhythms shown. (C) Diagram illustrating each step involved in the CEDIF (from transcription all the way to protein sorting in the Golgi) and RNA-Seq data for representative genes in XBP1^*Flox*^ and XBP1^*LKO*^ mice. Additional selected gene names belonging to each step are also shown. Data are graphed as the mean ± SEM (*n* = 2). *P* values indicating the robustness of 12-h rhythms detected by the RAIN method are shown for each gene in both XBP1^*Flox*^ (black) and XBP1^*LKO*^ (cyan) mice. (D) Clustering of genes involved in mRNA processing, ribosome biogenesis/translation initiation, and protein processing and transport based upon their superimposed 12-h transcriptome projected onto 3D t-SNE space in XBP1^*Flox*^ mice. (E) Polar histograms demonstrating phase distributions of genes involved in different steps of CEDIF in XBP1^*Flox*^ mice. (F) Scatter plot showing the phases of commonly found 12-h genes involved in different steps of CEDIF in XBP1^*Flox*^ and XBP1^*LKO*^ mice. Transcription is in cyan, mRNA processing in red, protein processing in the ER in blue, and Golgi apparatus in black. Numerical values are available in S2 Data, except for Figs 2D, the 3D scatter plot of which was automatically generated by Matlab. 3D, three-dimensional; CEDIF, central dogma information flow; CT, constant time; ER, Endoplasmic Reticulum; FPKM, Fragments Per Kilobase of transcript per Million mapped reads; GO, Gene Ontology; KEGG, Kyoto Encyclopedia of Genes and Genomes; RAIN, Rhythmicity Analysis Incorporating Nonparametric; RNA-Seq, RNA sequencing; t-SNE, t-distributed stochastic neighbor embedding; XBP1s, Spliced Form of X-box Binding Protein 1.

In sharp contrast to largely intact circadian rhythms in XBP1^*LKO*^ mice, ablation of XBP1 in the liver significantly impairs the global 12-h transcriptome profile (Fig 1K and S2C Fig). Specifically, we identified 2,501 (54.5%), 1,454 (31.6%), and 639 genes (13.9%), whose superimposed 12-h rhythms were abolished, dampened, or increased, respectively, in the absence of XBP1 (Fig 1K). For the 12-h genes commonly found in both XBP1^*Flox*^ and XBP1^*LKO*^ mice, we observed an average phase advance of 31 min in XBP1^*LKO*^ mice (Fig 1H). Subsequent GO analysis on either all 4,594 twelve-hour genes or those 3,955 (86.0%) genes whose 12-h rhythms were either abolished or dampened in XBP1^*LKO*^ mice revealed top enriched pathways of RNA polymerase II–mediated transcription, mRNA processing and surveillance, RNA export, translation and ribosome biogenesis, protein processing and sorting in the Endoplasmic Reticulum (ER) and Golgi apparatus, vesicle trafficking, and—to a lesser degree—immune pathway (Fig 2A–2C, S2F–S2I Fig, S4 and S6 Tables). The top enriched biological pathways are reminiscent of the progressive molecular processing sequence described by the central dogma information flow (CEDIF), namely, transcription and mRNA processing in the nucleus, ribosome biogenesis/translation in the nucleus and/or cytosol, and protein processing and sorting in the ER and/or Golgi apparatus in a temporal order (Fig 2A–2C). The GO analysis is robust to different GO category and background gene selections (S2F–S2I Fig). We further found that the 12-h rhythmic expression of genes involved in different steps of CEDIF are similarly affected by the ablation of XBP1s (S4 Table). Importantly, we found that both anabolism and catabolism pathways are enriched in both 12-h cycling mRNA and protein processing genes, suggesting an XBP1s-mediated overall 12-h rhythm of RNA and protein processing.

We subsequently focused on the XBP1s-dependent 12-h CEDIF genes. We divided CEDIF into 12 steps, carefully annotated the genes, and assigned each of them into one of the 12 steps (Fig 2C). We identified 54 genes involved in basal transcription regulation that include RNA polymerase II subunits (*Polr2b*, *Polr2h*), mediator complex subunits (*Med12*, *Med24*), and general transcription factor complex units (*Taf11*, *Taf12*, *Gtf2a2*). For genes involved in RNA metabolism (a total of 148 genes), they encompass pre-mRNA cleavage and polyadenylation, pre-mRNA splicing, RNA export, and mRNA degradation and nonsense-mediated mRNA decay, with prominent examples including cleavage stimulatory factor (*Cstf*) and cleavage and polyadenylation specificity factor (*Cpsf*) family members, numerous mRNA splicing factors and small nuclear ribonucleoproteins that are part of spliceosome (*Sf3a1*, *Snrpa*, *Snrpd1*, *Snrpe*), RNA export factors *Nxf1* and *Nxt1*, multipotent gene expression regulator Carbon Catabolite Repressor 4-Negative on TATA (CCR4-NOT) complex family member (*Cnot3*, *Cnot4*, *Cnot6*), and exosome complex components mediating RNA degradation (*Exosc1*, *Exosc3*) (Fig 2C and S6 Table). Overall, we found that the mean expression level of these genes was elevated in XBP1^*LKO*^ mice (Fig 2B).

The most dominant GO pathways are protein metabolism, which include a total of 393 genes that can be further categorized into 185 genes involved in protein processing in the ER and 208 genes in the Golgi apparatus (Fig 2B and 2C and S6 Table). These pathways include ER-associated protein degradation (*Edem1* and *Edem3*), translation initiation (eukaryotic initiation factor members), protein translocation across the ER membrane (the ER translocon *Sec61* members), protein folding in the ER (Heat Shock Protein 40 kD [HSP40] family members), protein glycosylation in the ER (multiple Asparagine-Linked Glycosylation [ALG] members), protein transport from the ER to the Golgi (Coatomer II [COPII] subunits *Sec13, Sec22*, and *Sec23*), Golgins family members (*Golga3–5*) playing key roles in the stacking of Golgi cisternae, and Golgi transport protein (*Golt1a* and *Golt1b*) involved in fusion of ER-derived transport vesicles with the Golgi complex. For these genes, the average expression level was lower in XBP1^*LKO*^ mice (Fig 2B).

To determine whether nuance exists in the 12-h transcriptome profile of genes involved in different steps of CEDIF, we performed t-distributed Stochastic Neighbor Embedding (t-SNE) analysis on the superimposed 12-h transcriptome of XBP1^*Flox*^ mice revealed by the eigenvalue/pencil method and revealed that the clustering of CEDIF genes by their superimposed 12-h transcriptome exhibits a spatial trajectory consistent with the direction of CEDIF (Fig 2D). Lastly, we observed a statistically significant progressive phase delay of genes involved in mRNA processing, protein processing in the ER, and protein sorting in the Golgi in XBP1^*Flox*^ mice, with an overall phase delay observed from CT1 and CT13 for mRNA processing genes to CT2 and CT14 for Golgi genes (Fig 2E). By contrast, no significant difference in phase distribution was observed between 12-h cycling transcription and mRNA processing genes in XBP1^*Flox*^ mice (Fig 2E), consistent with the known fact that pre-mRNA splicing occurs co-transcriptionally [16]. A similar progressive phase delay was not observed among 12-h genes in XBP1^*LKO*^ mice, and markedly different phase distributions of genes involved in different steps of CEDIF were also found between XBP1^*Flox*^ and XBP1^*LKO*^ mice (Fig 2E and 2F). This progressive phase delay observed in XBP1^*Flox*^ mice is again consistent with the unidirectional genetic information flow (Fig 2E). In sum, these data indicate a delicately orchestrated 12-h rhythm of CEDIF by XBP1s.

### Twelve-hour–clock regulation by XBP1s is robust to analytical method and threshold selection

**Fig 3:**
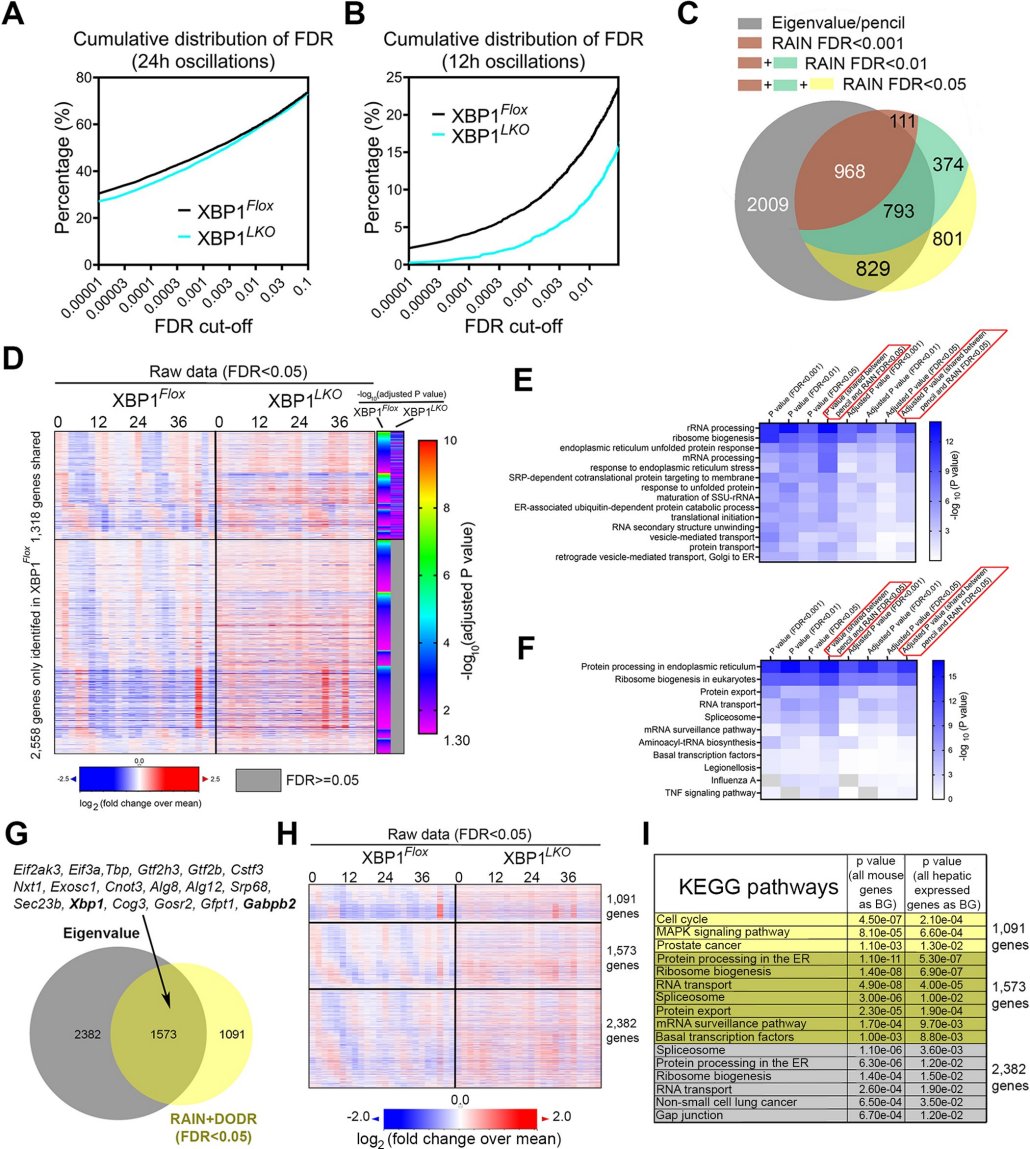
Prevalent XBP1s-dependent 12-h hepatic transcriptome revealed by RAIN. (A, B) Cumulative distribution of the percentage of circadian genes (A) or 12-h genes (B) under different FDR cut-offs in both XBP1^*Flox*^ and XBP1^*LKO*^ mice. (C) Venn diagram comparison of 12-h transcriptome uncovered by the eigenvalue and RAIN method (with different FDR cut-offs of 0.001, 0.01, and 0.05) in XBP1^*Flox*^ mice. (D) Heat map of the expression of 12-h cycling genes identified by RAIN. With an FDR < 0.05, 1,318 twelve-hour genes were commonly found in both XBP1^*Flox*^ and XBP1^*LKO*^ mice, and 2,558 twelve-hour genes were only identified in XBP1^*Flox*^ mice. Heat map showing the log_10_ transformed Benjamini-Hochberg procedure–adjusted *P* value for each identified 12-h gene was shown on the right. (E, F) Heat map summary of GO analysis demonstrating the log_10_ transformed *P* value of different enriched pathways under GOTERM_BP_DIRECT (panel E) and KEGG pathway (panel F) GO terms. GO was performed using all hepatic expressed genes as background. (G) Venn diagram comparison of XBP1s-dependent 12-h transcriptome uncovered by the eigenvalue and RAIN + DODR method (FDR < 0.05), with a short list of representative commonly identified genes. (H) Heat map of commonly and uniquely identified XBP1s-depenent 12-h transcriptome by the eigenvalue and RAIN methods. (I) GO analysis of commonly and uniquely identified XBP1s-depenent 12-h transcriptome by the eigenvalue and RAIN methods. Numerical values are available in S3 Data. BG, background; DODR, Detection of Differential Rhythmicity; ER, Endoplasmic Reticulum; FDR, False Discovery Rate; GO, Gene Ontology; KEGG, Kyoto Encyclopedia of Genes and Genomes; MAPK, Mitogen-Activated Protein Kinase; RAIN, Rhythmicity Analysis Incorporating Nonparametric; SRP, Signal Recognition Particle; SSU rRNA, small subunit ribosomal ribonucleic acid; TNF, tumor necrosis factor; XBP1s, Spliced Form of X-box Binding Protein 1.

To ensure that our discovery of the 12-h clock regulation by XBP1s is robust to different analytical methods and threshold selections, we further applied an alternative rhythm-identification algorithm, Rhythmicity Analysis Incorporating Nonparametric (RAIN) [17], to the hepatic RNA-Seq dataset (S6 Table). RAIN is a nonparametric method for the detection of rhythms of prespecified periods and of arbitrary wave forms and was previously found capable of robustly identifying both circadian and ultradian rhythms, although it can only identify one oscillation from each gene [6,7]. Consistent with the largely unaltered circadian rhythms in XBP1^*LKO*^ mice revealed by the eigenvalue/pencil method, similar numbers of circadian genes were identified under a wide range of FDR cut-offs in both XBP1^*Flox*^ and XBP1^*LKO*^ mice (Fig 3A). By contrast, compared to XBP1^*LKO*^ mice, XBP1^*Flox*^ mice are much more enriched with 12-h genes with small *P* values (a smaller *P* value indicates a much more robust 12-h rhythm) (Fig 3B). These results therefore demonstrate globally impaired 12-h rhythms, but not circadian rhythms, in XBP1^*LKO*^ mice, consistent with the results by the eigenvalue method.

A high congruence was found between the hepatic 12-h transcriptome uncovered by the eigenvalue and RAIN methods in XBP1^*Flox*^ mice, which became even more evident with more stringent FDR cut-offs with the RAIN method (Fig 3C and 3D, S3A and S3B Fig). At the FDR cut-off of 0.05, a total of 3,876 twelve-hour genes were identified in XBP1^*Flox*^ mice by the RAIN method, among which 2,558 genes were not found in XBP1^*LKO*^ mice (Fig 3D). GO analysis on the 12-h genes identified by the RAIN method (under different FDR cut-offs) and the 12-h genes commonly identified by both methods both revealed strongly enriched biological pathways of CEDIF as expected (Fig 3E and 3F). Because the RAIN method does not generate the exact amplitude of 12-h oscillation in each gene, we further performed DODR [18] analysis to detect differentially rhythmic 12-h oscillations in the 1,318 commonly found 12-h genes (FDR < 0.05) in both XBP1^*Flox*^ and XBP1^*LKO*^ mice and identified 106 twelve-hour genes that oscillate less robustly in XBP1^*LKO*^ mice with an FDR cut-off of 0.05 (S7 Table). Consequently, combining both RAIN and DODR analysis, with an FDR cut-off of 0.05, we identified a total of 2,664 XBP1s-dependent hepatic 12-h genes, of which 1,573 are commonly revealed by the eigenvalue method (Fig 3G and 3H). Not surprisingly, these 1,573 genes are very strongly enriched in CEDIF pathways and include canonical 12-h clock genes, including *Eif2ak3*, *Gfpt1*, *Alg12*, *Sec23b*, and *Xbp1* itself (Fig 3G–3I).

While a significant convergence of the identified 12-h transcriptome in XBP1^*Flox*^ mice was observed between the RAIN and eigenvalue methods, a 12-h transcriptome specifically identified by either one alone was also found (Fig 3C and 3G and S3C–S3I Fig). Many RAIN-specific 12-h transcriptomes were found to exhibit periods outside of the lower (10.5 h) and upper bound (13.5 h) of eigenvalue-defined 12-h rhythm (such as *Sec16b* and *Nphp3*) and thus was omitted by the eigenvalue analysis (S3D and S3E Fig). The eigenvalue-specific 12-h transcriptome, on the other hand, often contained superimposed circadian rhythms with larger amplitude (such as *Themis* and *Gm12718*) (S3F and S3G Fig) and thus was deemed not statistically significant to be considered 12-h rhythm by the RAIN method. Consequently, performing RAIN analysis on XBP1^*Flox*^ RNA-Seq data with all superimposed circadian rhythms removed identified a larger number of 12-h transcriptomes shared with the eigenvalue result (S3J Fig). Next, we compared the performance of the two methods in identifying 12-h transcriptomes that are abolished or dampened in XBP1^*LKO*^ mice (S3K–S3O Fig). Similar to the 1,573 commonly identified 12-h transcriptomes, 2,382 eigenvalue-specific XBP1s-dependent 12-h transcriptomes were also significantly enriched in CEDIF pathways (Fig 3H and 3I), many of which exhibited reduced amplitude in XBP1^*LKO*^ mice but did not reach statistical significance of FDR < 0.05 by the DODR analysis (such as *Sec61a1* and *Ddx55*) (S3N and S3O Fig). By contrast, the 1,091 RAIN-specific 12-h genes were not enriched in any CEDIF pathways, but rather showed enriched pathways of cell cycle and Mitogen-Activated Protein Kinase (MAPK) signaling (Fig 3I and S3K–S3M Fig). However, eigenvalue analysis of such genes as *Cdk2* and *Dusp10* did not reveal any superimposed 12-h oscillations, but rather a combination of oscillations with various other periods (S3L and S3M Fig).

Because many 12-h cycling oscillations are superimposed with other oscillations with larger amplitude (circadian oscillations, for example) (S4A and S4B Fig), we went on to confirm that these “non-dominant” 12-h rhythms are also subject to XBP1s-dependent 12-h clock regulation. We first compared the 12-h transcriptome identified from RAIN (FDR < 0.05) with all the 4,594 12-h genes and the 1,709 dominant 12-h genes identified by the eigenvalue method. In fact, under the FDR cut-off of 0.05, 1,428 “non-dominant” 12-h genes can be identified by the RAIN method (S4C Fig). GO analysis revealed that these 1,428 genes are also enriched in CEDIF pathways (S4D Fig). Taking the *Sec63* gene as an example, in XBP1^*Flox*^ mice, the eigenvalue/pencil method showed that it has a larger circadian rhythm superimposed with a smaller 12-h rhythm. In XBP1^*LKO*^ mice, only the circadian rhythm was identified. Similar results were also validated by the RAIN method (in XBP1^*Flox*^ mice, the *P* value for detecting a circadian and a 12-h rhythm was 1.04 × 10^−11^ and 0.0020, respectively; in XBP1^*LKO*^ mice, the *P* value for detecting a circadian and a 12-h rhythm was 5.44 × 10^−11^ and 0.869, respectively) (S4E Fig). These results indicate that XBP1s regulates the 12-h—but not the circadian—rhythm of *Sec63* expression, even though the 12-h oscillation is not the dominant one. Globally, of the 1,428 genes, the 12-h rhythms of 889 genes were not identified in the XBP1^*LKO*^ mice by the RAIN method (FDR < 0.05) (S4F Fig). For the 1,458 “non-dominant” 12-h–rhythm genes that are specifically identified by the eigenvalue method, they are enriched in a number of immune pathways as well as splicesomes (S4G Fig). For instance, *Setd1b* has superimposed 12-h and 24-h rhythms in XBP1^*Flox*^ mice. In XBP1^*LKO*^ mice, the 12-h rhythm was abolished, while the circadian rhythm remained intact (S4H Fig). Globally, of the 1,458 genes, the 12-h rhythms of 818 genes were not identified in the XBP1^*LKO*^ mice by the eigenvalue method (S4I Fig). These results demonstrate that the “non-dominant” 12-h rhythms are still under XBP1s-dependent 12-h clock control. In sum, notwithstanding some disparity between the 12-h transcriptome revealed by the two methods, the discovery of prevalent XBP1s-dependent 12-h transcriptomes—especially those involved in CEDIF—is robust to statistical methods and threshold choices.

### Cell-autonomous 12-h rhythms of CEDIF gene expression in hepatocytes and MEFs

We next addressed the question of whether the 12-h rhythms of gene expression observed in vivo are also cell autonomous. First, we performed a post hoc analysis of time series transcriptome of serum-synchronized murine liver MMH-D3 cells [19] (see Materials and methods for details) using the eigenvalue method. Similar to what is observed in vivo, the majority of oscillations identified in MMH-D3 cells were circadian rhythms (which cycle at a slightly shorter period of 21.6 h, with an FDR of 0.08) and oscillations that cycle at the second (approximately 10.8 h, FDR = 0.22) and third harmonics (approximately 7.3 h) of the circadian rhythm (S5A and S5B Fig, and S8 Table). Genes with circadian oscillations were enriched in biological pathways of circadian rhythm and Hypoxia-Inducible Factors (HIF-1) signaling and include core circadian clock genes (S3C–S3E Fig and S8 Table), consistent with past findings [19–22]. The eigenvalue-method–identified 12-h transcriptomes were further confirmed by the RAIN method (S9 Table).

**Fig 4:**
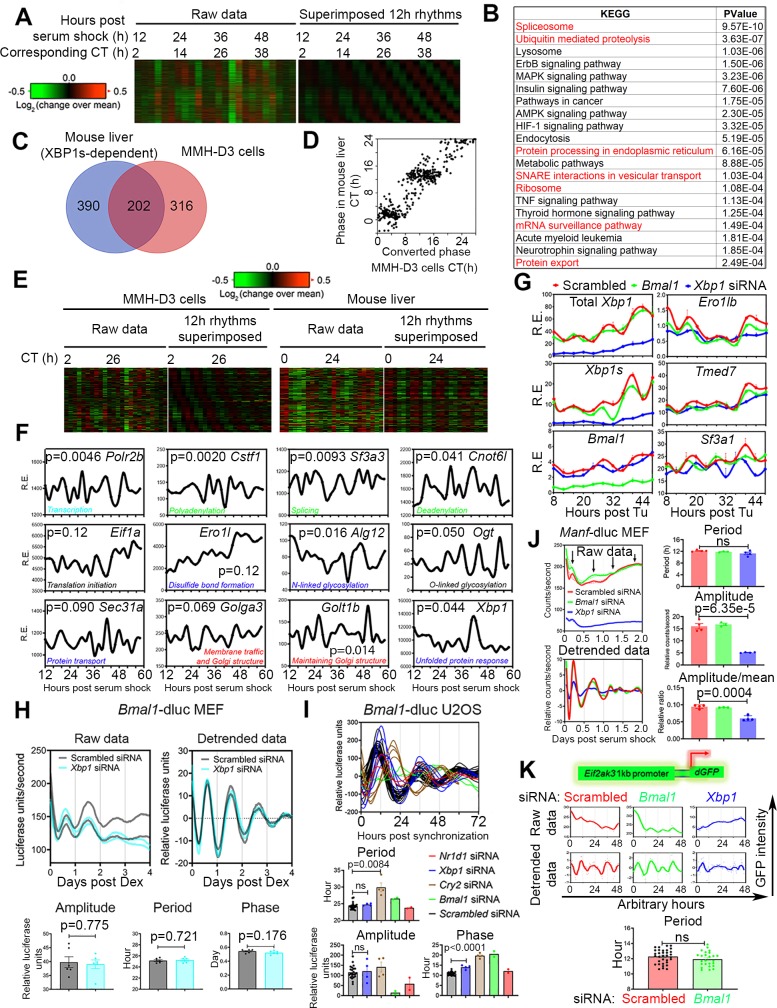
The 12-h rhythms of gene expression are cell autonomous. (A) Heat map of all 12-h cycling gene expression uncovered from serum shock-synchronized murine liver cell line MMH-D3 identified by the eigenvalue/pencil analysis [19], with both raw data and superimposed 12-h rhythms shown. Both the original time after serum shock as well as the CT are shown. (B) GO analysis showing enriched KEGG pathways and their corresponding *P* values for cell-autonomous 12-h transcriptome, with GO terms related to CEDIF highlighted in red. (C) Venn diagram comparison of 12-h transcriptome involved in CEDIF from mouse liver in vivo and MMH-D3 cells in vitro. (D) Scatter plot comparing phases of CEDIF-related 12-h gene oscillation in MMH-D3 cells and mouse liver. The phases of 12-h oscillations in MMH-D3 are converted to CT. (E) Heat map of side-by-side comparison of CEDIF-related 12-h gene expression in MMH-D3 cells and mouse liver, with both raw data and superimposed 12-h rhythms shown. (F) Microarray data of representative 12-h cycling genes involved in CEDIF in MMH-D3 cells compiled from [19]. *P* values indicating the robustness of 12-h rhythms detected by RAIN are also shown for each gene. (G) MEFs were transfected with different siRNAs and treated with Tu (25 ng/ml) for 2 h, and qPCR was performed at different times post Tu shock. Data are graphed as the mean ± SEM (*n* = 3–9). (H) Real-time luminescence analysis of *Bmal1-dluc* MEFs post 100 nM Dex treatment. Representative raw and detrended traces of luminescence recordings from MEFs subject to different siRNA transfection (top) and quantified amplitude, period, and phases (bottom). (I) Real-time luminescence traces of *Bmal1-dluc* U2OS cells transfected with different siRNAs as reported in [23] (top) and quantified amplitude, period, and phases (bottom). (J) Real-time luminescence analysis of *Manf-dluc* MEFs post 50% horse serum shock. Raw and detrended traces of luminescence recordings from MEFs subject to different siRNA transfection (left) and calculated period, amplitude, and mean-normalized amplitude (right). (K) Representative recordings (top) and period quantification (bottom) of single-cell time-lapse microscopy analysis of *Eif2ak3* promoter-driven dGFP oscillation in scrambled siRNA, *Bmal1* siRNA, or *Xbp1* siRNA transfected MEFs. Data are graphed as the mean ± SEM. Numerical values are available in S4 Data. AMPK, 5' AMP-activated protein kinase; CEDIF, central dogma information flow; CT, constant time; Dex, dexamethasone; dGFP, destabilized green fluorescent protein; GO, Gene Ontology; HIF, Hypoxia-inducible factor; KEGG, Kyoto Encyclopedia of Genes and Genomes; MAPK, Mitogen-Activated Protein Kinase; MEF, mouse embryonic fibroblast; MMH-D3, Met murine hepatocytes-3 days old; ns/NS, not significant; qPCR, quantitative PCR; RAIN, Rhythmicity Analysis Incorporating Nonparametric; siRNA, small interfering RNA; SNARE, soluble nsf attachment protein receptor; TNF, tumor necrosis factor; Tu, tunicamycin; XBP1s, Spliced Form of X-box Binding Protein 1.

Enriched biological pathways associated with 12-h genes (9.5 h to 12.5 h) in MMH-D3 cells reveal large convergence with those found in mouse liver in vivo, including all CEDIF as well as several immune pathways (Fig 4A–4C, S5F–S5K Fig, S10 and S11 Tables). Specifically, 202 twelve-hour CEDIF genes were commonly found in mouse liver in vivo and in MMH-D3 cells in vitro (Fig 4C–4F, S10 and S11 Tables), which also include the 12-h oscillation of total *Xbp1* mRNA expression (Fig 4F). After converting the time post serum shock into CT time, we further observed a similar bimodal phase distribution of those 12-h CEDIF genes at dawn and dusk (Fig 4D and 4E). For the other 390 twelve-hour CEDIF genes only found in MMH-D3 cells in vitro, they often represent different members of the same gene family as found in vivo (S5K Fig). While the phases of 12-h CEDIF genes were also largely enriched around dawn and dusk (at CT11 and CT23) in MMH-D3 cells, compared to their in vivo counterparts, they exhibited a larger variance (S5L Fig). In addition, 12-h genes involved in mRNA processing and protein processing in the ER and Golgi apparatus in these cells did not exhibit a statistically significant progressive phase delay as observed in vivo (S5L Fig). This discrepancy could be due to the technical aspects of the original microarray data and data analysis (e.g., lack of replicates of the raw microarray data, introduction of bias during data detrending, etc.), or it could imply the existence of non–cell-autonomous factors that also contribute to the 12-h clock phase control.

To determine whether the 12-h and circadian clocks are also independently regulated in a cell-autonomous manner in vitro, we knocked down *Xbp1* or *Bmal1* using siRNA and performed qPCR in dexamethasone- or tunicamycin-synchronized MEFs as previously described [6]. In agreement with the unaffected core circadian clock in XBP1^*LKO*^ mice liver, knocking down *Xbp1* does not diminish dexamethasone-synchronized circadian oscillations of *Bmal1*, *Per2*, or *Reverbα* (*Nr1d1*) expression, whose rhythms are nonetheless significantly impaired by *Bmal1* knockdown as expected (Fig 4G and S5M Fig). To more quantitatively evaluate the effects of 12-h clock knockdown on the circadian clock period, phase, and amplitude, we knocked down *Xbp1* and performed real-time luminescence on MEFs stably expressing *Bmal1* promoter-driven destabilized luciferase. Under both dexamethasone and serum shock conditions, knocking down *Xbp1* did not alter the period, the amplitude, or the phase of *Bmal1* oscillation, to a level that reached statistical significance (*P* < 0.05) (Fig 4H and S5N Fig). Post hoc analysis of a previously published dataset [23] further demonstrated that *Xbp1* knocking down did not change the circadian period or the amplitude of *Bmal1* oscillation in human U2OS cells, although a slight phase delay of *Bmal1* oscillation was observed in this study post *Xbp1* knocking down (Fig 4I). This discrepancy in phases could be due to different cell lines and synchronization methods used.

While *Xbp1* knocking down does not affect the circadian clock in vitro, it significantly impairs the cell-autonomous 12-h CEDIF gene expression in tunicamycin-synchronized MEFs, which are not affected by *Bmal1* knockdown (Fig 4G). Consistent with the in vivo result, robust 12-h rhythms of both total and spliced *Xbp1* expression were observed, with a similar phase difference of 3.5 h found between the two (Fig 4G). We next confirmed that horse serum shock can indeed entrain the 12-h clock by performing real-time luminescence on MEFs stably expressing *Manf* promoter-driven destabilized luciferase (*Manf*-dluc) (*Manf* gene encodes an ER-resident/secreted protein that is under 12-h clock–control [6]) and found that *Xbp1s* but not *Bmal1* knockdown significantly reduces both the basal expression as well as the amplitude (both raw and mean-normalized) of 12-h luciferase oscillation (Fig 4J). Single-cell time-lapse imaging using MEFs stably expressing *Eif2ak3*-destabilized green fluorescent protein (dGFP) [6] further demonstrated that *Xbp1*, but not *Bmal1*, regulates cell-autonomous 12-h rhythms of gene expression (Fig 4K). This evidence strongly supports the existence of cell-autonomous 12-h rhythms of CEDIF gene expression.

One intriguing observation from analyzing the periods of all harmonic oscillations in MMH-D3 cells is that these oscillations seem to be period-locked to the circadian rhythm (7.3 h × 3 = 21.9 h; 10.8 h × 2 = 21.6 h) (S5A Fig). To determine whether the period of approximately 12-h oscillation is also 1:2 locked to that of the circadian rhythm in cells with altered circadian period, we analyzed a previously published metabolome dataset in dexamethasone-synchronized U2OS cells [24]. In agreement with the results shown in Fig 4J and 4K, knocking down *Bmal1* did not alter the period of 12-h–oscillating metabolites while almost completely ablating the circadian metabolites (S5O Fig). Intriguingly, while *Cry2* knocking down lengthened the period of circadian metabolites in U2OS cells as expected, it failed to affect the period of 12-h–oscillating metabolites (S5O Fig). These results strongly demonstrate that the 12-h and circadian periods are independent of each other at the cellular level. Based upon these data, we hypothesize that the period locking of 12-h and circadian rhythms observed in MMH-D3 cells likely indicates that the two clocks can adjust their periods concordantly with a locked 1:2 ratio in the presence of varying environmental changes. As previous studies showed that the circadian period will change in response to pH, oxygen, and temperature alterations [21,25,26], it is equally possible that the period of 12-h clock can also change accordingly. Future studies are needed to further test this hypothesis.

### GA-binding proteins as putative novel transcription regulators of the mammalian 12-h clock

**Fig 5:**
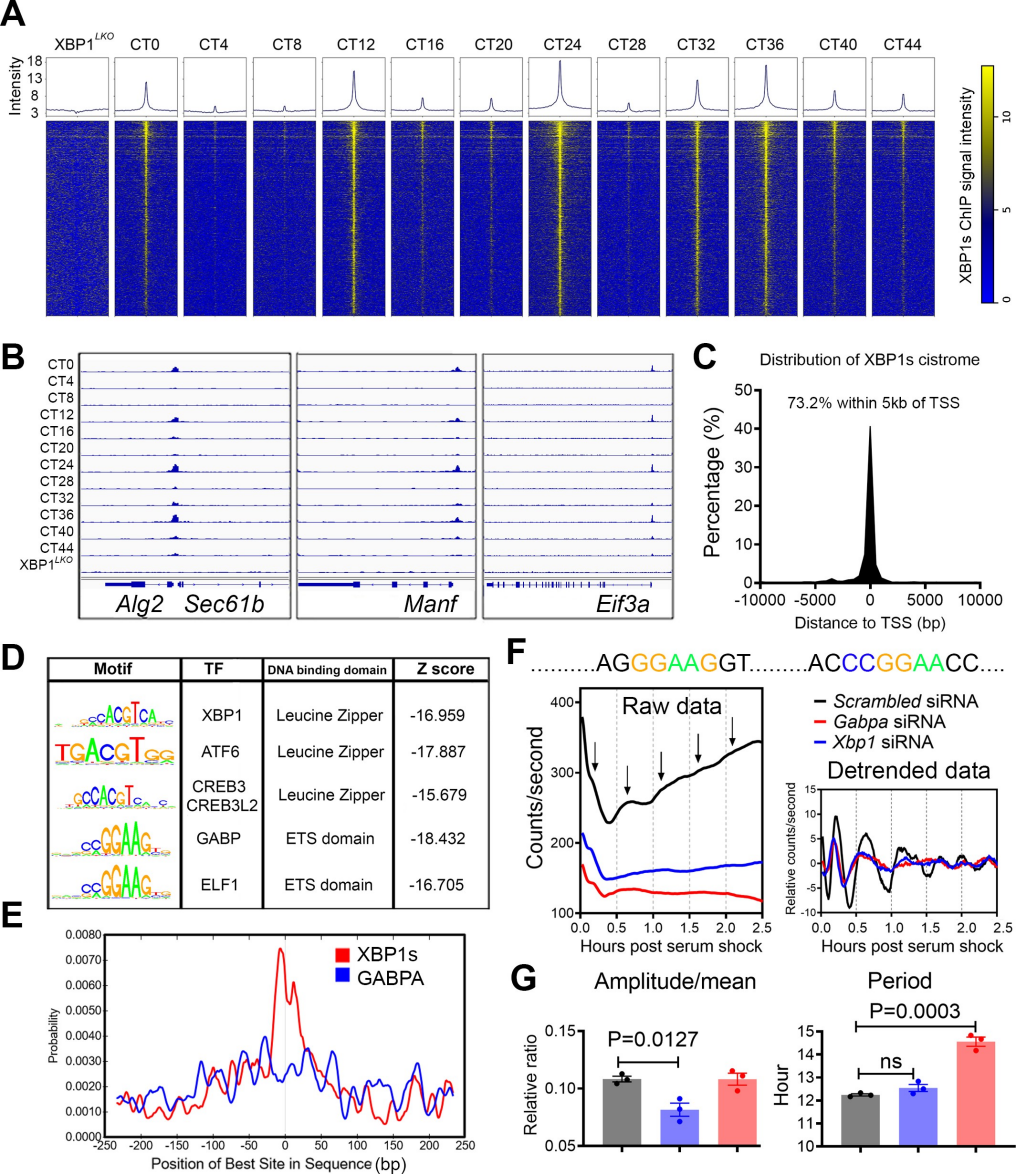
GABPA is a putative new transcriptional regulator of the mammalian 12-h clock. (A) Heat maps of all XBP1s binding signal at 4-h intervals in XBP1^*Flox*^ mice as well as in XBP1^*LKO*^ mice surrounding the center of XBP1s binding sites (±2 kb). (B) Snapshot of target genes selected for alignment of hepatic XBP1s binding sites at different CTs in XBP1^*Flox*^ and XBP1^*LKO*^ mice. (C) Distribution of the distance between XBP1s binding sites and the TSS. (D) Top enriched SeqPos motifs common to 12-h cycling XBP1s cistrome. (E) Position distribution of XBP1s or GABPA motifs relative to XBP1s peak center. (F, G) Real-time luminescence analysis of *Manf-dluc* MEFs post 50% horse serum shock. Consensus GABPA binding sites in the promoter of mouse *Manf* gene, raw and detrended traces of luminescence recordings from MEFs subject to different siRNA transfection (F) and quantification of relative amplitude and period (G). Data are graphed as mean ± SEM. Numerical values are available in S12 Table and S4 Data except for Fig 5E, which was automatically generated by CentriMo toolbox (version 5.1.0) (http://meme-suite.org/tools/centrimo). ATF6, Activating Transcription Factor 6; CREB3, Cyclic AMP-Responsive Element-Binding protein 3; CT, constant time; ELF1, E74 Like ETS Transcription Factor 1; GABPA, GA-binding protein; MEF, mouse embryonic fibroblast; SeqPos, Sequence Position; siRNA, small interfering RNA; TF, transcription factor; TSS, transcription start site; XBP1s, Spliced Form of X-box Binding Protein 1.

Having established that XBP1s contributes to the establishment of 12-h rhythms of mRNA expression, we next set out to investigate the detailed molecular mechanisms by which it occurs. XBP1s is known to act as a basic region leucine zipper (bZIP) transcription factor activating gene expression by binding to gene regulatory regions harboring consensus DNA sequence GCCACGT under ER stress condition [27–29]. Since hepatic XBP1s expression exhibits a 12-h rhythm under physiological conditions without exogenous ER stress at both the mRNA and protein level (Fig 1A–1C) [3,6], we performed hepatic XBP1s chromatin immunoprecipitation sequencing (ChIP-Seq) (at a 4-h interval for a total of 48 h) to globally profile its cistrome under constant darkness condition and identified a total of 1,681 high confidence binding sites (see Materials and methods for details) (Fig 5A and S12 Table). Consistent with its oscillating expression, XBP1s cistrome cycles with a 12-h period, with peak binding observed at CT0, CT12, CT24, and CT36 (Fig 5A and 5B). Consistent with the independent relationship between the circadian and 12-h clocks, very limited overlap of core circadian clock transcription factors’ cistromes is observed with that of XBP1s (S6A Fig).

The hepatic XBP1s cistrome is predominantly enriched around the proximal promoter, compared to other regions of the genome (Fig 5C and S6B–S6D Fig), which is consistent with previously reported XBP1s cistrome distribution in human triple-negative breast cancers [28]. Very intriguingly, we found highly enriched XBP1s binding sites at bidirectional promoter regions (S6C and S6D Fig). Gene pairs regulated by XBP1s-targeted bidirectional promoters exhibit similar XBP1s-dependent 12-h oscillation of expression (S6C Fig). XBP1s transcriptional regulation of bidirectional promoters may allow for a more tightly coordinated temporal control of the mammalian 12-h clock [30].

Motif analysis of the XBP1s cistrome reveals enriched motifs associated with leucine zipper- containing transcription factor binding sites as expected, including XBP1, Activating Transcription Factor 6 (ATF6), and Cyclic AMP-Responsive Element-Binding protein 3 (CREB3)/Cyclic AMP-Responsive Element-Binding protein 3-Like (CREB3L) (Fig 5D). Both ATF6 and CREB3/CREB3L1/CREB3L2 are known to activate gene expression involved in UPR [31–34], and ATF6 and CREB3L2 also exhibit 12-h rhythms of gene expression (S6E Fig), with 12-h rhythm of the active form of ATF6 (p60) protein level also confirmed by western blot (S6F Fig) [6]. These data suggest that ATF6 and CREB3L2 may cooperate with XBP1s in dictating 12-h rhythms of gene expression at the transcriptional level. In addition to leucine zipper transcription factors, we unexpectedly found enriched motifs of E26 Transformation-Specific (ETS) transcription factors, including GA-binding protein (GABP) and E74 Like ETS Transcription Factor 1 (ELF1) at the XBP1s cistrome (Fig 5D). While the ETS transcription factors are known to play important roles in tissue development and cancer progression [35,36], to the best of our knowledge, their potential involvement in the regulation of CEDIF and cross-talk with XBP1s remains unreported. Compared to XBP1s DNA binding motif, GABP binding sites are distributed more diffusely around XBP1s peak center (Fig 5E). This result indicates that GABP and/or ELF1 binding sites most likely occur adjacent to that of XBP1s *in cis*. GABP transcriptionally regulates gene expression predominantly by forming a heterotetrameric complex composed of 2 α and 2 β subunits encoded by the *Gabpa* and *Gabpb1/b2* genes, respectively [37]. Both hepatic *Gabpa* and *Gabpb2* mRNA exhibit XBP1s-depenent 12-h oscillations (Fig 3G and S6G Fig), and 12-h chromatin recruitment of XBP1s to *Gabpa* gene promoter was also found (S6H Fig). By examining a previously published temporal hepatic proteome database [38], we found a robust 12-h oscillation of nuclear GABPA level (S6I Fig). In addition, robust 12-h oscillations of nuclear GABPA/GABPB2 bound to an artificial DNA fragment harboring GABP consensus sequence were also reported in the same study (S6I Fig) [38]. Contrary to the phase of XBP1s expression, GABPA protein level peaks at CT6 and CT18 (S6I Fig). To determine whether GABPA regulates 12-h rhythm of gene expression, we utilized the *Manf-dluc* MEF cell line. Motif analysis of the *Manf* promoter revealed 2 GABPA DNA binding motifs (Fig 5F). siRNA-mediated knockdown of *Gabpa* lowered the basal level of the luciferase signal but did not alter the relative amplitude of *Manf*-dluc oscillation. Instead, it significantly lengthened the period of 12-h luciferase oscillation (Fig 5F and 5G). While future efforts are needed to firmly establish the causal roles of GABP in transcriptionally regulating the mammalian 12-h rhythms of gene expression, these data nevertheless imply the putative concerted actions of GABP with XBP1s in regulating the mammalian 12-h clock (see Discussion section for further details).

### The motif stringency of XBP1s promoter binding sites dictates XBP1s’s ability to drive 12-h rhythms of transcription of CEDIF genes

**Fig 6:**
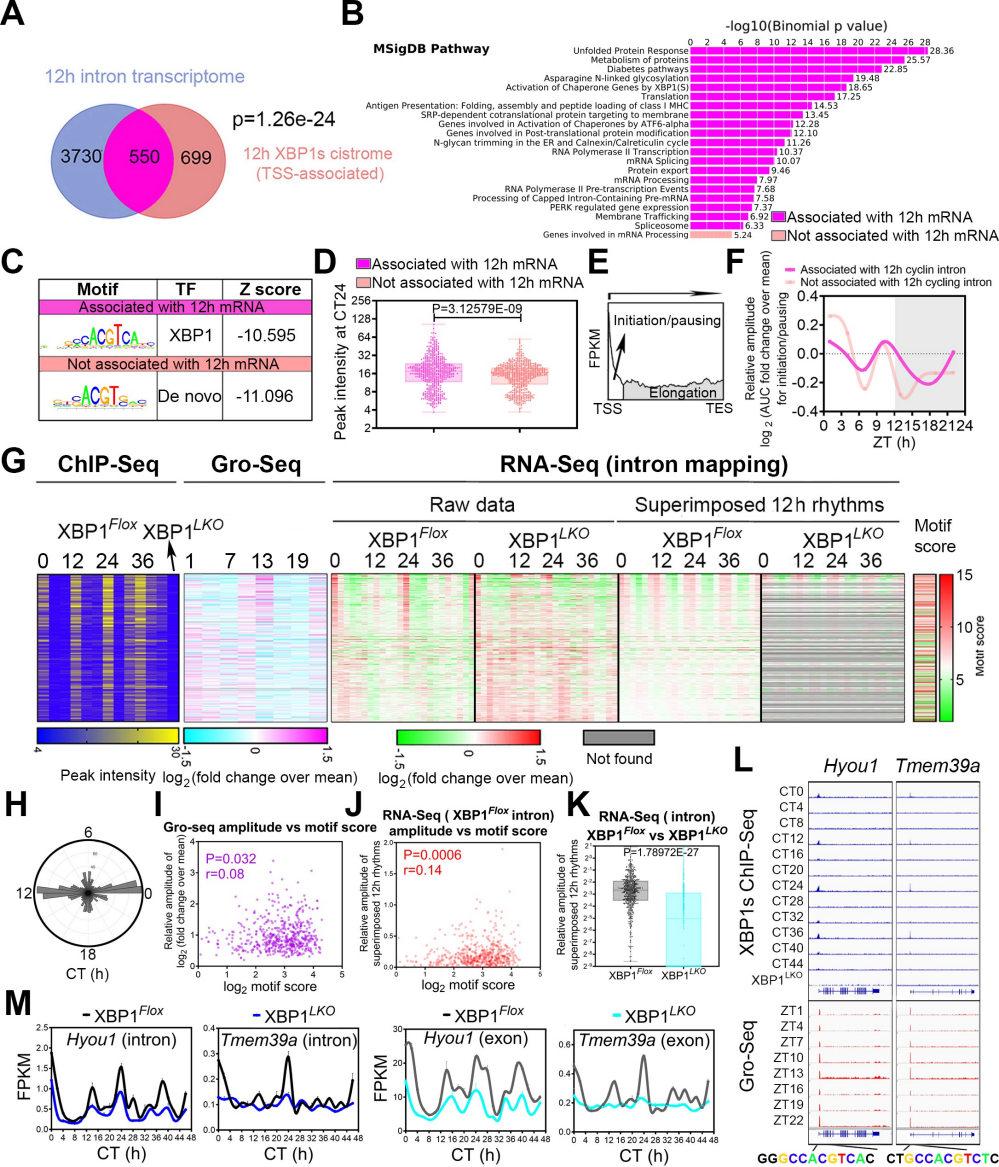
The motif stringency of XBP1s binding sites underlies the 12-h rhythms of mRNA transcription. (A) Venn diagram depicting common and unique intron-mapping 12-h cycling and TSS XBP1s-binding genes. (B) GO analysis showing enriched MSigDB pathways and their corresponding *P* values for 550 genes with both intron-mapping 12-h cycling transcriptome and TSS XBP1s binding (purple) and 699 genes with TSS XBP1s binding but no intron-mapping 12-h cycling transcriptome (beige). (C) Top enriched SeqPos motifs common to XBP1s cistromes with or without associated intron-mapping 12-h cycling transcriptome. (D) Calculated peak intensity of XBP1s binding at CT24 for XBP1s cistrome associated with or without intron-mapping 12-h cycling transcriptome. (E) A representative diagram depicting a typical Gro-Seq signal from TSS to TES of a gene and using AUC to calculate both transcription initiation/pausing and transcription elongation rates. (F) Log_2_ mean-normalized transcription initiation rates calculated from the Gro-Seq data [42] for TSS XBP1s target genes with or without associated intron-mapping 12-h transcriptome. (G–L) 550 genes with both proximal promoter XBP1s binding and intron-mapping 12-h transcriptome in XBP1^*Flox*^ mice. (G) Heat maps of XBP1s binding intensity, transcription initiation rates calculated from the Gro-Seq [42], intron-mapping 12-h cycling gene expression (or lack thereof) in XBP1^*Flox*^ and XBP1^*LKO*^ mice with both raw data and superimposed 12-h rhythms shown, and XBP1s binding motif score. (H) Polar histogram demonstrating the phase distributions of 550 intron-mapping 12-h cycling genes with TSS XBP1s binding in XBP1^*Flox*^ mice. (I) Scatter plot of Log_2_ transformed XBP1s binding motif score versus the relative amplitude of mRNA transcription initiation rates calculated from the Gro-Seq [42] in XBP1^*Flox*^ mice, together with Spearman’s rank correlation coefficient *r*, and the *P* value at which *r* is significantly different than 0. (J) Scatter plot of Log_2_ transformed XBP1s binding motif score versus the relative amplitude of superimposed intron-mapping 12-h rhythms in XBP1^*Flox*^ mice, together with Spearman’s rank correlation coefficient *r*, and the *P* value at which *r* is significantly different than 0. (K) Relative amplitude of intron-mapping 12-h rhythms for each gene in XBP1^*Flox*^ and XBP1^*LKO*^ mice. If a 12-h rhythm is not found in XBP1^*LKO*^ mice, then an amplitude of 0 is used. (L) Snapshot of target genes selected for alignment of hepatic XBP1s binding sites at different CTs in XBP1^*Flox*^ and XBP1^*LKO*^ mice as well as published Gro-Seq data [42]. Consensus XBP1s binding motifs identified at each gene promoter are also shown. (M) RNA-Seq data (both intron and exon mapping) for representative genes in XBP1^*Flox*^ and XBP1^*LKO*^ mice. Data are graphed as the box and whisker plot (minimum to maximum) in panels D and K and mean ± SEM in panel M. Numerical values are available in S4 Data. AUC, area under the curve; ChIP-Seq, Chromatin Immunoprecipitation Sequencing; CT, constant time; FPKM, Fragments Per Kilobase of transcript per Million mapped reads; GO, Gene Ontology; Gro-Seq, Global run-on Sequencing; MHC, major histocompatibility complex; MSigDB, Molecular Signatures Database; PERK, Protein kinase R-like Endoplasmic Reticulum Kinase; RNA-Seq, RNA sequencing; SeqPos, Sequence Position; SRP, Signal Recognition Particle; TES, transcription termination site; TF, transcription factor; TSS, transcription start site; XBP1s, Spliced Form of X-box Binding Protein 1.

We focused our subsequent analysis on proximal promoter XBP1s binding sites as they can be unambiguously assigned to each gene and thus permit identification of genes exhibiting both 12-h cycling mRNA and 12-h XBP1s cistrome. Compared to total mRNA expression, pre-mRNA have been shown to provide better proxies for transcription regulation due to their short half-life [39–41]. We therefore estimated the pre-mRNA level of each transcript by quantifying intron-mapping reads from the RNA-Seq dataset and identified 4,280 twelve-hour–cycling intron-mapping transcripts in XBP1^*Flox*^ mice (Fig 6A and S13 Table). Comparison of 12-h pre-mRNA transcriptome and XBP1s cistrome revealed a moderate overlap, which reaches statistical significance, indicating an enrichment of XBP1s target genes with 12-h rhythm of pre-mRNA expression (Fig 6A). The 3,730 twelve-hour genes without XBP1s binding were strongly enriched in mRNA processing pathways (S6J Fig). Motif analysis on the promoters of these genes confirmed the lack of XBP1s consensus binding motif but instead revealed strong enrichment of GABP and ELF1 binding sequences (S6K Fig). Furthermore, the 12-h phases of these genes are heavily enriched at CT6 and CT18 (S6L Fig), matching those of GABP protein oscillations (S6I Fig). These data strongly suggest that GABP may drive 12-h rhythms of gene expression with more diverse phases.

We were surprised to find 699 genes with XBP1s binding near promoters but without 12-h pre-mRNA oscillation and eager to identify the potential mechanisms that distinguish these 699 genes (cistrome positive) from the 550 genes (double positive) with both 12-h XBP1s cistrome and transcriptome (Fig 6A). GO analysis revealed that while the 550 double-positive genes (including *Eif2ak3*, *Manf*, *Sec61a*, and *Xbp1* itself) are strongly enriched in CEDIF pathways as expected, the 699 cistrome-positive genes lack strongly enriched GO categories (Fig 6B and S14 Table). Motif analysis of the promoters of double-positive genes reveals expected XBP1s consensus motif CCACGTCA (Fig 6C). Very intriguingly, the top enriched motif for the cistrome-positive gene promoters is identified as a de novo motif with the sequence (C/G)ACGT(G/C), which resembles a degenerate XBP1s motif (Fig 6C). XBP1s binding intensity to the degenerate motif is weaker compared with the binding to the consensus XBP1s sequence (Fig 6D). Accordingly, XBP1s binding to the double-positive—but not the cistrome-positive—gene promoters is strongly associated with the 12-h rhythm of nascent pre-mRNA transcription initiation (but not elongation) peaking at CT0 and CT12, as assayed by both calculating the area under the curve of the pre-pausing portion of Global run-on Sequencing (Gro-Seq) signal [42] (Fig 6E and S6M Fig) and our intron-mapping RNA-Seq data (Fig 6F–6M and S6N Fig). Overall, for the 550 double-positive genes, we observed a positive correlation among the amplitude of nascent pre-mRNA oscillation (assayed by Gro-Seq), the amplitude of pre-mature mRNA oscillation (assayed by intron-mapping reads), and the XBP1s binding motif stringency score (assayed by ChIP-Seq and the higher the score, the more similar the XBP1s binding motif is to the consensus sequence) in XBP1^*Flox*^ mice (Fig 6G–6M).

Taken together, we hereby demonstrated that the ability of XBP1s to drive 12-h rhythms of mRNA transcription is strongly influenced by the motif of its DNA binding sites: while 12-h XBP1s chromatin recruitment to gene promoters harboring consensus XBP1s DNA binding motif CCACGTCA is strongly associated with its ability to drive 12-h rhythms of transcription initiation, weaker XBP1s binding to promoters with degenerate motifs is much less likely to lead to robust 12-h gene oscillations (S6O–S6R Fig).

### The 12-h rhythms of gene expression are evolutionarily conserved in marine animals possessing circatidal clock

**Fig 7:**
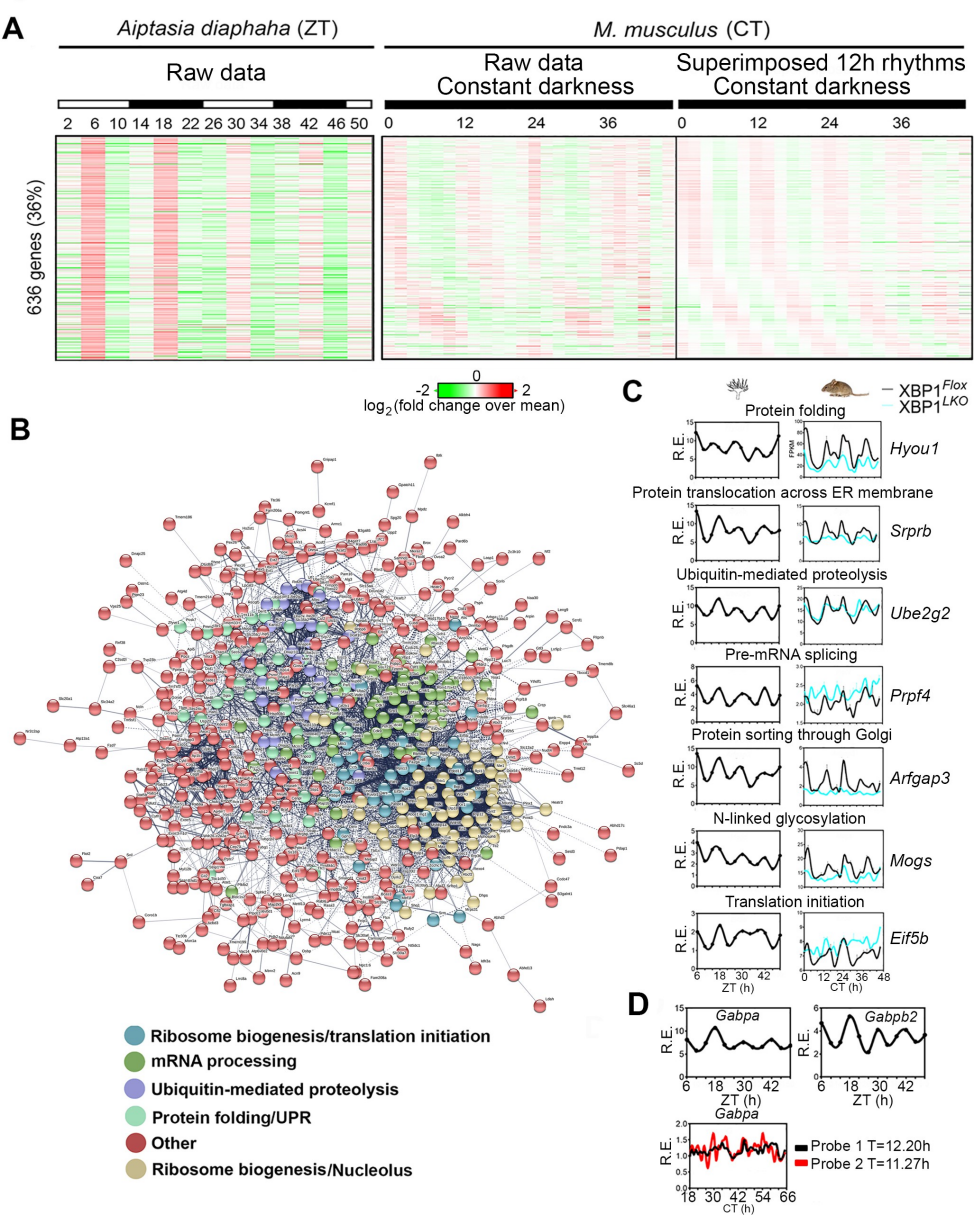
The 12-h rhythms of CEDIF gene expression are evolutionarily conserved in circatidal animals. (A) Heat map of side-by-side comparison of evolutionarily conserved 12-h gene expression in aposymbiotic *A*. *diaphaha* [48] and mouse liver, with both raw data and superimposed circatidal rhythms shown for the mouse liver data. (B) Predicted interactive network construction of these conserved 12-h cycling genes using STRING [49]. Genes involved in different biological pathways are colored differently. (C) RNA-Seq data for representative genes in *A*. *diaphaha* [48] and XBP1^*Flox*^ and XBP1^*LKO*^ mouse liver. Data are graphed as the mean ± SEM (*n* = 2). (D) *Gabpa* and *Gabpb2* expression in aposymbiotic *A*. *diaphaha* [48] (top) and *Gabpa* expression in mouse liver from the 48-h microarray dataset [2] indicated by 2 different probes. Numerical values are available in S4 Data. A higher-resolution image of panel B is available in S2 Raw images. CEDIF, central dogma information flow; CT, constant time; R.E., relative expression; RNA-Seq, RNA sequencing; STRING, Search Tool for the Retrieval of Interacting Genes/Proteins; UPR, Unfolded Protein Response; XBP1, X-box Binding Protein 1; ZT, Zeitgeber time.

We originally hypothesized that the mammalian 12-h clock evolved from the circatidal clock of coastal and estuarine animals that modulates their behavior in tune with the ebb and flow of the tides with an approximately 12.4-h period [1,6], which were also shown to be driven by a dedicated circatidal pacemaker distinct from the circadian clock [43–47]. To seek further support for our hypothesis, we analyzed 2 recently published time series RNA-Seq datasets of 2 marine animals exhibiting a circatidal clock, aposymbiotic sea anemone *Aiptasia diaphaha* [48] and the limpet *Cellana rota* [10]. In both cases, we found a large overlap of 12-h cycling transcripts between mouse and these two tidal species (Fig 7A–7C, S6A–S6C Fig, and S15 Table). Computationally constructing predicted interactive networks using the overlapping 12-h cycling genes in both species via Search Tool for the Retrieval of Interacting Genes/Proteins (STRING) [49] or by traditional GO analysis revealed sub-hubs involved in different steps of CEDIF (Fig 7B, S6B Fig, and S16 Table). In addition, we found that the spliceosome is within the top two enriched KEGG GO terms in the circatidal transcriptomes of the 2 marine animals (regardless of their conservation status in mice) (S16 Table). We further observed 12-h oscillations of *Gabpa* in both *A*. *diaphaha* and *C*. *rota* and of *Gabpb2* in *A*. *diaphaha* (Fig 7D and S6D Fig). Twelve-hour oscillation of *Xbp1* in *C*. *rota* was previously reported [1]. These data combined with the recent independent report of an earlier evolutionary origin of genes cycling with a 12-h period [8] provide strong support for our hypothesis that the mammalian 12-h clock evolved from the circatidal clock.

## Discussion

### The eigenvalue/pencil versus RAIN method

In this study, we applied eigenvalue/pencil and RAIN methods to detect 12-h transcriptome. Apart from the commonly found 12-h transcriptome, we further observed 12-h transcriptome that is uniquely identified by each method. This discrepancy can be largely contributed to the distinct nature of the two methods. Most of the current rhythm-identification methodologies (such as RAIN, JTK_CYCLE, and ARSER) require the user to define a narrow period range and then use respective algorithms to identify one oscillation from each temporal dataset that minimizes the *P* value [7]. Because the *P* value of each identified oscillation is known, the users are given the freedom to select the FDR threshold of their choice. The eigenvalue method, on the other hand, presumes that each biological time series dataset consists of multiple superimposed oscillations (a fact also highlighted by a recent study examining ultradian rhythms in *Neurospora* [50]) and thus permits unbiased identification of all superimposed oscillations without any constraints on the period, amplitude, or phases. As such, the *P* value information for each identified oscillation is not available, and only one fixed FDR can be obtained for all identified oscillations through a permutation-based method. While a strong oscillation (which alone can largely account for the waveform of the original temporal dataset) can be detected by both methods with high confidence (such as *Alg12* and *Sec23b*), a weaker oscillation (which is superimposed by other oscillations with similar or larger amplitudes) often evades detection (and is mistaken for different periods) by the RAIN method (such as *Cdk2* and *Dusp10*). Nevertheless, there always remains the question of whether such weaker oscillations with small amplitudes are a consequence of technical artifacts or of real biological significance. This problem can often be satisfactorily addressed by cross-validation with additional data from independent sources and/or technical platforms. Overall, the eigenvalue/pencil method is more sensitive in detecting weaker ultradian oscillations but at the same time has a higher type I error (higher false positive rate), while the RAIN method is more stringent and consequently has a higher type II error (higher false negative rate). We thus recommend that the eigenvalue/pencil method be used first as a discovery tool to identify all potential oscillations, followed by RAIN analysis to solidify the conclusion.

### The working model of the transcriptional regulation of the mammalian 12-h clock by XBP1s

**Fig 8:**
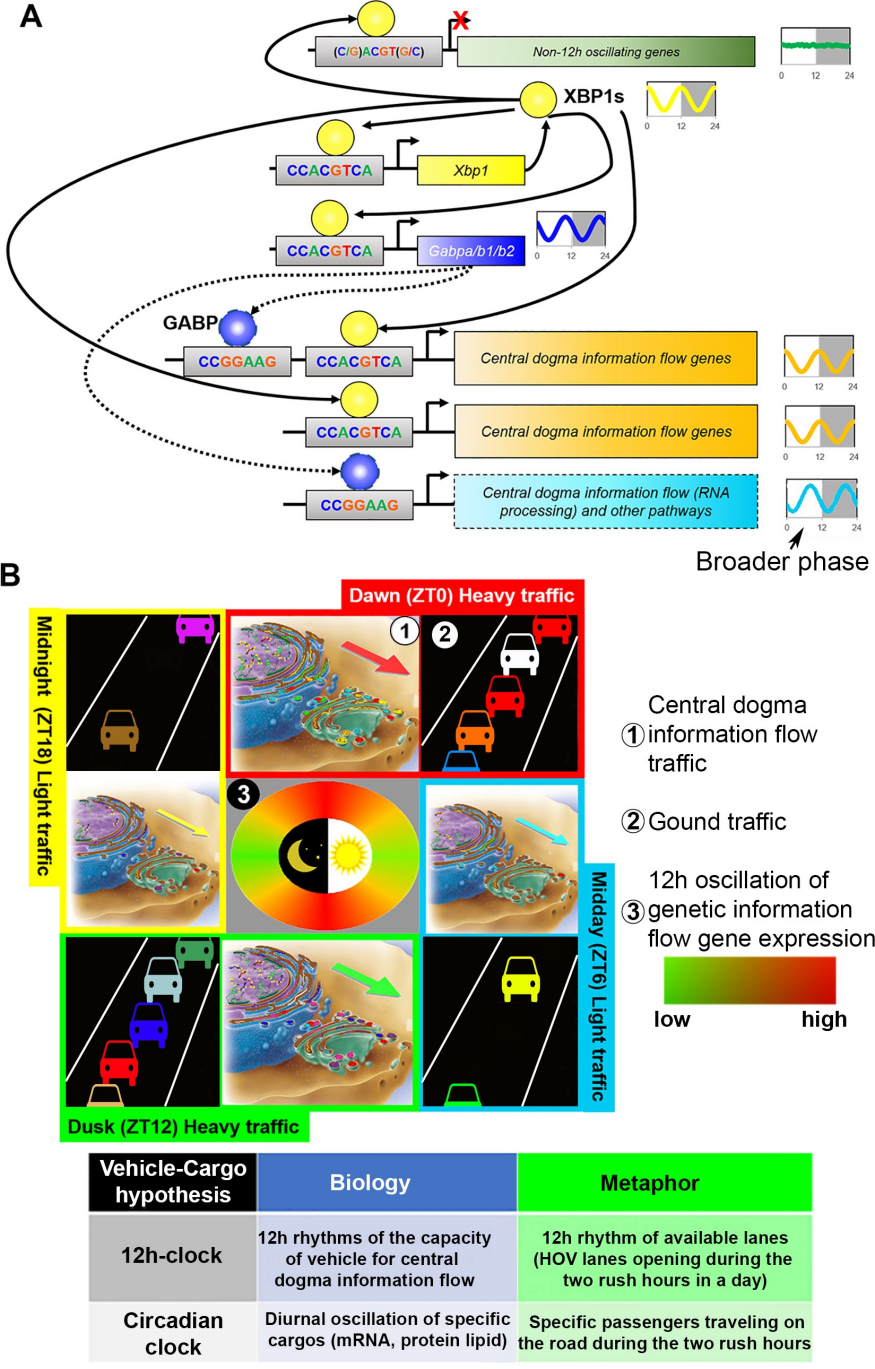
XBP1s transcriptionally regulates 12-h rhythms of gene expression involved in CEDIF. (A) A simplified model summarizing our current understanding of the transcriptional regulation of the mammalian 12-h clock by XBP1s. Twelve-hour rhythmic XBP1s binding to consensus XBP1s binding motif CCACGCTA within proximal promoter regions drives 12-h rhythms of gene expression that are involved in regulating the traffic capacity of CEDIF. XBP1s further self-regulates its own 12-h gene expression via this mechanism, thus forming a positive feedforward loop. On the other hand, 12-h rhythmic XBP1s binding to degenerate XBP1s binding motif fails to drive 12-h rhythms of gene expression. XBP1s further transcriptionally regulates 12-h oscillation of GABP transcription factors, whose binding motif exhibits equal strong enrichment on the promoters of XBP1s-dependent 12-h genes. (B) The vehicle-cargo hypothesis on the distinct functions of 12-h clock versus the 24-h circadian clock. Similar to the increased traffic at “rush” hours at each dawn and dusk in people’s daily life, 12-h rhythms of CEDIF gene expression peaking at dawn (CT0) and dusk (CT12) (indicated by the circular heat map in the middle) suggest the existence of a 12-h oscillation of “traffic” of CEDIF, which consists of progressive molecular processing steps from transcription, mRNA processing, ribosome biogenesis, and translation, all the way to protein processing/sorting in the ER and Golgi. At this point, it remains to be determined whether it is the total number of molecules undergoing processing (illustrated by the varying arrow sizes) or the metabolic rate of processing (or both) that exhibits a 12-h oscillation. The vehicle-cargo hypothesis posits this: whereas the 12-h clock regulates the 12-h rhythms of the traffic capacity of the CEDIF (thus the vehicle), the circadian clock (and other temporal and tissue-specific mechanisms) contributes to the regulation of diurnal oscillations of specific cargos that undergo molecular processing. CEDIF, central dogma information flow; CT, constant time; ER, Endoplasmic Reticulum; GABP, GABPA, GA-binding protein; HOV, high-occupancy vehicle; XBP1s, Spliced Form of X-box Binding Protein 1; ZT, Zeitgeber time.

Our study establishes XBP1s as a major transcriptional regulator of the mammalian 12-h clock. Hepatic ablation of XBP1 results in the impairment of 86% (by eigenvalue method) or 69% (by RAIN method) of hepatic 12-h cycling transcriptome. XBP1s directly transcriptionally regulates more than 500 genes (with acrophases around CT0 and CT12) via rhythmic binding to promoters containing consensus sequence CCACGTCA. Alternatively, XBP1s transcriptionally regulates 12-h oscillations of GABP expression, which in turn can bind to gene promoters harboring ETS consensus sequence CCGGAAG and putatively regulates additional 12-h transcriptome with a wider range of acrophases (particular at CT6 and CT18). GABP can also act in concert with XBP1s *in cis* on a subset of genes containing adjoining XBP1s and GABP DNA binding motifs in gene promoters (Fig 8A). XBP1s also regulates its own 12-h transcription, thus completing a positive feedforward loop (Fig 8A) [51]. At this point, what remains elusive is the mechanism(s) of negative feedback required for sustaining cell-autonomous oscillations of the 12-h clock. One potential candidate is the unspliced form of XBP1 (Xbp1us), which has been previously shown to negatively regulate UPR by forming a complex with XBP1s and ATF6 in the cytosol and targeting them for ubiquitin-mediated degradation [52,53]. One last note is that what is summarized in Fig 8 is the current working model of the transcriptional regulation of the mammalian 12-h clock by XBP1s; it is subject to revision and modification with more available experimental and mathematical modeling data in the future. For instance, it is equally likely that GABPA can act upstream of XBP1s and transcriptionally regulates 12-h rhythm of *Xbp1s* expression and subsequent 12-h cycling output genes. In this case, XBP1s becomes the mediator between GABPA and 12-h cycling output genes. Future work focused on temporal transcriptome profiling in GABPA liver-specific knockout mice will distinguish between these two models.

As an important note, the data presented herein do not rule out the possibility that the 12-h rhythms of some genes may be influenced by the circadian clock and/or the effects of certain external cues as previously suggested [3,5]. Furthermore, although our data indicate that the 12-h clock is independent from the circadian clock at the cellular level, they appear to be able to cross-talk at the systemic metabolic level [4]. Therefore, future studies are needed to investigate the physiologic conditions by which these distinct clocks can interact systemically in model organisms.

### The vehicle-cargo hypothesis on the distinct functions of 12-h clock versus the circadian clock

A very fascinating finding from our study is the coordinated 12-h oscillations of genes involved in the entire CEDIF, ranging from mRNA transcription, mRNA processing and export, and translation regulation to protein processing and sorting in the ER and Golgi, which include both anabolic and catabolic processes. The vast majority of these genes peak at dawn (CT0 to CT3) and dusk (CT12 to CT15), corresponding to the transition periods between fasting/feeding and rest/activity that are associated with elevated metabolic stress [1]. In light of these findings, we hereby propose a vehicle-cargo hypothesis that attempts to decipher the distinct functions of the 12-h clock versus the circadian clock (Fig 8B). We argue that the 12-h clock accommodates rush hours’ (at dawn and dusk) elevated gene expression and processing by controlling the 12-h rhythms of the global traffic capacity (and/or the traffic rate) of the CEDIF (thus the vehicle), in tune with the 12-h cycle of metabolic stress [1] (Fig 8B). The circadian clock and/or other tissue-specific pathways, on the other hand, dictate the particular genes or gene products processed at each rush hour (thus the cargo) as previously suggested [54] (Fig 8B). An everyday metaphor would be the fluctuating daily traffic on the highway: the 12-h clock is analogous to the highway that increases its operating capacity during the morning and evening rush hours (by opening the high-occupancy vehicle [HOV] lane, for example), while the 24-h circadian clock is likened to dictating the specific cars that go on the highway during morning and evening (Fig 7B). Future efforts should be directed toward characterizing the temporal profile of mRNAs and proteins being processed in response to the 12-h clock in the nucleus and the ER/Golgi, respectively.

## Materials and methods

### Ethics statement

The animal studies were carried out in accordance with the National Institutes of Health guidelines and were granted formal approval by the University of Pittsburgh’s Institutional Animal Care and Use Committee (approval number IS00013119 and IS00013119) and BCM’s Institutional Animal Care and Use Committee (approval number AN-544).

### Cell lines

MEF was prepared as previously described [14]. *Manf*-dluc MEF was generated by cloning a 750-bp (−569 bp to +181 bp) promoter region of mouse *Manf* gene into pGL4.16[luc2CP/Hygro] vector (E6711; Promega), which encodes for a destabilized luciferase gene (forward primer: TAGGCAGGCCGAGACCTTTCGTTTA; reverse primer: ATAACTAGTCTTTCGCTTTCCTTGGGTTTAG) and subsequently transfecting the vector into MEFs and selecting for stable clones in the presence of 200 μg/ml hygromycin. *Bmal1*-dluc MEF was generated by lentiviral infection of MEFs with pLV6-Bmal-luc-packaged lentivirus, and stable clones were selected by culturing in the presence of 10 μg/ml blasticidin. pLV6-Bmal-luc was a gift from Steven Brown (Addgene [https://www.addgene.org/] plasmid #68833; http://n2t.net/addgene:68833; RRID:Addgene_68833).

### Animals

XBP1^*Flox*^ mice were previously described [11]. XBP1^*LKO*^ mice were generated by crossing Albumin-CRE mice with XBP1^*Flox*^ mice. All mice are of C57BL/6 background, male, and between 3 and 4 mo of age. Mice were first entrained under LD12:12 conditions for 2 wk before being transferred to constant darkness for 24 h. Mice were then euthanized at 2-h intervals for a total of 48 h. Mice were fed ad libitum during the entire experiment.

### Food intake monitoring

Comprehensive Lab Animal Monitoring System (CLAMS) Calorimetry (Columbus Instruments, http://www.colinst.com/) was used for real-time measuring of food intake. XBP1^*Flox*^ (*n* = 4) and XBP1^*LKO*^ (*n* = 4) mice were acclimated to the chambers for at least 1 wk, and ad libitum food intake was monitored for 72 h under LD12:12 followed by 96 h of constant darkness.

### Locomotor activity monitoring

The Home Cage Activity System (Omnitech Electronics, http://www.omnitech-electronics.com/) was used for real-time measuring of spontaneous locomotor activity in a home-cage environment. XBP1^*Flox*^ (*n* = 3–4) and XBP1^*LKO*^ (*n* = 3–4) mice were acclimated to the home cage for at least 1 wk and fed ad libitum. Spontaneous locomotor activity was measured under either LD12:12 condition or constant-darkness conditions.

### siRNA transient transfections

MEFs were transfected with 10 μM of different siRNAs for approximately 24 to 48 h with Lipofectamine RNAiMAX reagents (Life Technologies, https://www.thermofisher.com/us/en/home.html) per the manufacturer’s instructions. Sources of siRNA (from Horizon Discovery, https://horizondiscovery.com/) are as follows: siGENOME Non-Targeting siRNA pool (Dharmacon, D-001206-1305), siGENOME SMARTpool ARNTL (Dharmacon, L-040483-01-0005), siGENOME SMARTpool XBP1 (Dharmacon, L-040825-00-0005), and siGENOME SMARTpool GABPA (Dharmacon, L-041036-01-0005).

### Synchronization of MEFs

MEFs were isolated from male SRC-2^*fl/fl*^ mice and immortalized by SV40 T antigen as previously described [14]. For tunicamycin treatment, MEFs were cultured in DMEM (4.5 g/L glucose) supplemented with 10% FBS, treated with 25 ng/ml of tunicamycin for 2 h, and then washed with 1X PBS before cultured in the same medium. For dexamethasone treatment, MEFs were cultured in DMEM (4.5 g/L glucose) supplemented with 10% FBS, treated with 100 nM dexamethasone for 30 min, and then washed with 1X PBS before cultured in the same medium. For all cell culture experiments, cells were cultured at 37°C with 5% CO_2_. For serum shock, MEFs were cultured in DMEM (4.5 g/L glucose) supplemented with 10% FBS, treated with 50% horse serum for 2 h, and then washed with 1X PBS before cultured in the same medium.

### Real-time luminescence assay

Stable *Manf*-dluc or *Bmal1*-dluc MEFs were cultured in DMEM (4.5 g/L glucose) supplemented with 10% FBS and treated with 50% horse serum in DMEM for 2 h or 100 nM for 30 min before being subjected to real-time luminescence assay using a Lumicycle (Actimetrics, https://www.actimetrics.com/) as previously described [55]. Briefly, after serum shock treatment, MEFs were washed with 1X PBS and cultured with DMEM (4.5 g/L glucose) supplemented with 0.1 mM Luciferin and 10 mM HEPES buffer in 35-mm tissue culture dishes in the absence of serum and were transferred immediately to Lumicycle for real-time luminescence analysis. Periods of oscillation were identified by embedded Periodogram function. For siRNA-treated MEFs, MEFs were transfected with nontargeting or *Bmal1*, *Gabpa*, or *Xbp1* siRNA for 48 h before being subjected to serum shock and real-time luminescence assay as described earlier.

### Time-lapse microscopy

Time-lapse microscopy was performed on *Eif2ak3*-dGFP cells transfected with scrambled, *Bmal1*, or *Xbp1* siRNAs using IncuCyte Live Cell Analysis System (Essen Bioscience, https://www.essenbioscience.com/en/) as previously described [6]. *Eif2ak3*-dGFP was subject to imaging using 300 ms integration time at 30-min interval. During the imaging, the cells were cultured in serum-free medium. GFP intensity for single cells (cell lineages) was subsequently quantified automatically by automated image processing software CellProfiler (version 2.2.0, https://cellprofiler.org/) [56]. The raw data were subjected to polynomial detrend (*n* = 4–7), and the eigenvalue/pencil method was applied to uncover top superimposed oscillations from mathematically detrended data.

### Immunoblot

Immunoblot analyses were performed as described previously [57]. Briefly, proteins separated by 4% to 20% gradient SDS-PAGE gels (Bio-rad, https://www.bio-rad.com/) were transferred to nitrocellulose membranes, blocked in TBST buffer supplemented with 5% bovine serum albumin (BSA), and incubated overnight with primary anti-XBP1s antibody (Biolegend Poly6195, https://www.biolegend.com/), anti-ATF6 antibody (Santa Cruz 22799, http://www.scbt.com), and anti-BMAL1 antibody (Abcam 3350, https://www.abcam.com/) at 4°C. Blots were incubated with an appropriate secondary antibody coupled to horseradish peroxidase at room temperature for 1 h, reacted with ECL reagents per the manufacturer’s (Thermo, https://www.thermofisher.com/us/en/home.html) suggestion, and were detected on X-ray film by autoradiography.

### qRT-PCR

Total mRNA was isolated from MEFs with PureLink RNA mini kit (Thermo) per the manufacturer’s instructions. Reverse transcription was carried out using 5 μg of RNA using Superscript III (Thermo) per the manufacturer’s instructions. For gene expression analyses, cDNA samples were diluted 1/30-fold (for all other genes except for 18S RNA) and 1/900-fold (for 18S RNA). qPCR was performed using the Taqman or SYBR green system with sequence-specific primers and/or the Universal Probe Library (Roche, https://www.roche.com/). All data were analyzed with 18S or *β-actin* as the endogenous control. qPCR primer sequences are as follows:

Mouse total *Xbp1* forward primer: gggtctgctgagtcc

Mouse total Xbp1 reverse primer: cagactcagaatctgaagagg

Mouse *Xbp1s* forward primer: ccgcagcaggtgc

Mouse *Xbp1s* reverse primer: cagactcagaatctgaagagg

Mouse *Xbp1us* forward primer: actatgtgcacctctgcag

Mouse *Xbp1us* reverse primer: cagactcagaatctgaagagg

Mouse *Arntl* forward primer: gccccaccgacctactct

Mouse *Arntl* reverse primer: tgtctgtgtccatactttcttgg

Mouse *Nr1d1* forward primer: acgaccctggactccaataa

Mouse *Nr1d1* reverse primer: ccattggagctgtcactgtaga

Mouse *Per2* forward primer: caacacagacgacagcatca

Mouse *Per2* reverse primer: tcctggtcctccttcaacac

Mouse *Ero1lb* forward primer: atgattcgcaggaccacttt

Mouse *Ero1lb* reverse primer: tcagcagcaggtccacatac

Mouse *Sf3a1* forward primer: gatgatgaggtttatgcaccag

Mouse *Sf3a1* reverse primer: agtacgtcgctcagccaact

Mouse *Tmed7* forward primer: agttggagaagacccaccttt

Mouse *Tmed7* reverse primer: agagcttcatgaatggaaacg

Mouse 18s RNA forward primer: ctcaacacgggaaacctcac

Mouse 18s RNA reverse primer: cgctccaccaactaagaacg

Mouse *β-actin* forward primer: aaggccaaccgtgaaaagat

Mouse *β-actin* reverse primer: gtggtacgaccagaggcatac

### RNA-Seq

Mouse liver tissues were collected from XBP1^*Flox*^ (*n* = 2) and XBP1^*LKO*^ (*n* = 2) mice at 2-h intervals for a total of 48 h under constant-darkness condition. Total RNA was isolated from mouse liver with miRNeasy Mini Kit (Qiagen, https://www.qiagen.com/us/) per the manufacturer’s instructions. Extracted RNA samples underwent quality control assessment using the RNA Pico 6000 chip on Bioanalyzer 2100 (Agilent, https://www.agilent.com/) and were quantified with Qubit Fluorometer (Thermo Fisher, https://www.thermofisher.com/us/en/home.html). Strand-specific total mRNA-Seq libraries were prepared using the Universal Plus mRNA-Seq kit (NuGen, https://www.nugen.com/) per the manufacturer’s instructions using 200 ng of RNA. The size selection for libraries were performed using SPRIselect beads (Beckman Coulter, https://www.beckman.com/), and purity of the libraries was analyzed using the High Sensitivity DNA chip on Bioanalyzer 2100 (Agilent). The prepared libraries were pooled and sequenced using NoveSeq 6000 (Illumina, https://www.illumina.com/), generating an average of 40 million paired-end reads of 2 × 100 bp length per sample (S1 Table). RNA-Seq library preparation and sequencing were performed at UPMC Genome Center (https://ipm.pitt.edu/UGC). Raw RNA-Seq FASTQ files were analyzed by FastQC (https://www.bioinformatics.babraham.ac.uk/projects/fastqc/) for quality control. Adaptors and low-quality reads were filtered by Trimmomatic (http://www.usadellab.org/cms/?page=trimmomatic) [58]. Then, the processed reads were aligned by HISAT2 (https://ccb.jhu.edu/software/hisat2/index.shtml) [59] against mouse reference mm10. Gene and isoform FKPM values were calculated by Cufflinks (http://cole-trapnell-lab.github.io/cufflinks/). For gene-level intron quantification, BEDTools software (https://bedtools.readthedocs.io/en/latest/content/tools/intersect.html) [60] was used to collect and count reads that aligned to any intron of the given gene. If one read spanned across multiple exons of the same gene, it would only be counted once. The intron counts were normalized by gene length and total reads for FPKM normalization.

### Identification of oscillating transcriptome

Averaged FPKM values at each time were used for cycling transcripts identification. Superimposed oscillations for the remaining transcripts were identified using the previously described eigenvalue/pencil method [6,7]. Specifically, 3 oscillations were identified from each gene. The criteria for circadian genes are as follows: period between 21 h and 25 h, decay rate between 0.8 and 1.2, and mean expression FPKM larger than 0.1; for approximately 12-h genes: period between 10.5 h to 13.5 h, decay rate between 0.8 and 1.2, and mean expression FPKM larger than 0.1; for approximately 8-h genes: period between 7 h and 9 h, decay rate between 0.8 and 1.2, and mean expression FPKM larger than 0.1. The mean value and standard deviation of circadian and approximately 12-h oscillations were determined by fitting 2 normal distributions to the experimental data, and the results are provided in Tab 1 of S3 Table. The relative amplitude is calculated by dividing the amplitude by the mean expression value of each gene. We determined the background gene expression level of FPKM = 0.1 by randomly selecting 70 genes that are known to be not expressed in the liver (or at least not expressed to a level deemed to be physiologically important). These genes include 50 keratin family members preferentially expressed in the skin, 19 genes preferentially expressed in the neurons, and the *adiponectin* gene preferentially expressed in the adipocyte. The average expression of these 70 genes is 0.092, and we rounded up to 0.1. To determine the FDR of identification of rhythmic transcripts, we used a permutation-based method that randomly shuffles the time label of gene expression data and subjected each of the permutation datasets to the eigenvalue/pencil method applied with the same criteria [13]. These permutation tests were run 10,000 times, and the FDR was estimated by taking the ratio of the mean number of rhythmic profiles identified in the permutated samples (false positive ones) to the number of rhythmic profiles identified in the original data. All the analyses were performed in MatlabR2017A (https://www.mathworks.com/?s_tid=gn_logo). Heat maps were generated by Gene Cluster 3.0 (http://bonsai.hgc.jp/~mdehoon/software/cluster/) and TreeView 3.0 alpha 3.0 (https://bitbucket.org/TreeView3Dev/treeview3/src/master/) using log2 mean-normalized values. The sorting of heat maps in Fig 1J and 1L was performed by averaging the phases between the two genotypes for each gene and ranked from small to large phases (phase delay from top to bottom). RAIN analysis was performed as previously described in Bioconductor (3.4) (http://www.bioconductor.org/packages/release/bioc/html/rain.html) [17], and DODR analysis was performed as previously described in R package [18].

### Defining oscillating genes

For the eigenvalue method, every gene consists of multiple superimposed oscillations. Therefore, we define a circadian gene as any gene that exhibits a superimposed circadian rhythm, regardless of its relative amplitude compared to other superimposed oscillations. Similar definitions apply to 12-h and 8-h genes. Under this definition, a gene can be both a circadian and 12-h cycling gene. By comparison, we define a dominant circadian gene as any gene whose superimposed circadian rhythm has the largest amplitude among all oscillations. Similar definitions also apply to 12-h and 8-h genes. Under this definition, dominant circadian and dominant 12-h genes are mutually exclusive.

### t-SNE analysis

t-SNE analysis was performed on identified pure 12-h oscillations using MatlabR2017A using the “tsne” function. The “Exact” algorithm and “Euclidean” distance metric were used. The three-dimensional scatter graph was generated with the “scatter3” function.

### Statistical test on phase segregation

The Watson-Wheeler (or Mardia-Watson-Wheeler, or uniform score) test was performed to detect the difference among the phase distributions of different groups of genes. The difference between the samples can be in either the mean or the variance.

### ChIP-Seq

ChIP for XBP1s was performed using anti-XBP1s antibody (Biolegend Poly6195) as previously described [57]. Briefly, mouse liver samples were submerged in PBS + 1% formaldehyde, cut into small (approximately 1 cm^3^) pieces with a razor blade, and incubated at room temperature for 15 min. Fixation was stopped by the addition of 0.125 M glycine (final concentration). The tissue pieces were then treated with a TissueTearer and finally spun down and washed twice in PBS. Chromatin was isolated by the addition of lysis buffer, followed by disruption with a Dounce homogenizer. The chromatin was enzymatically digested with MNase. Genomic DNA (Input) was prepared by treating aliquots of chromatin with RNase and proteinase K and heated for reverse-crosslinking, followed by ethanol precipitation. Pellets were resuspended, and the resulting DNA was quantified on a NanoDrop spectrophotometer. An aliquot of chromatin (30 μg) was precleared with protein A agarose beads (Invitrogen, https://www.thermofisher.com/us/en/home.html). Genomic DNA regions of interest were isolated using 4 μg of antibody. Complexes were washed, eluted from the beads with SDS buffer, and subjected to RNase and proteinase K treatment. Crosslinking was reversed by incubation overnight at 65°C, and ChIP DNA was purified by phenol-chloroform extraction and ethanol precipitation. The DNA libraries were prepared at the University of Pittsburgh and sequenced at Gene by Gene (https://genebygene.com/) per standard protocols. DNA libraries were prepared with Ovation Ultralow V2 DNA-Seq library preparation kit (NuGen) using 1 ng input DNA. The size selection for libraries was performed using SPRIselect beads (Beckman Coulter), and purity of the libraries was analyzed using the High Sensitivity DNA chip on Bioanalyzer 2100 (Agilent). The prepared libraries were pooled and sequenced using Nova-Seq 6000 (Illumina), generating approximately 30 million 75-bp single-end reads per sample.

### ChIP-Seq analysis

Replicates were pooled at each time point for subsequent ChIP-Seq analysis. The sequences identified were mapped to the mouse genome (UCSC mm10) using the “BOWTIE” function in Galaxy Deeptool (https://usegalaxy.org/) [61]. Only the sequences uniquely mapped with no more than 2 mismatches were kept and used as valid reads. PCR duplicates were also removed. Peak calling was carried out by MACS2 (version 2.1.1.20160309) in Galaxy (https://usegalaxy.org/) (options—mfold 5, 50—*P* = 1 × 10^−4^), on each ChIP-Seq file against the ChIP-Seq of XBP1^*LKO*^ mice. To account for the different sequencing depths between samples, the BAM files generated from MACS2 were RPKM normalized to sequencing depth using the bamCoverage function in Galaxy Deeptool (https://usegalaxy.org/), and the bigwig files were generated accordingly. The relative intensity of each XBP1 binding site is further calculated via the computeMatrix function with the RPKM normalized bigwig files and bed files from the peak calling as inputs by calculating the area under the curve. Due to the robust 12-h oscillation of XBP1s hepatic nuclear proteins [3,6], only XBP1s cistromes whose binding intensity exhibits robust 12-h oscillations (period between 10.5 h and 13.5 h; decay rate between 0.8 and 1.2; phase between 0 h and 3 h) are selected as bona fide XBP1s binding sites.

### GO analysis

DAVID (version 6.8) [62] (https://david.ncifcrf.gov) and GREAT (version 3.0.0, http://great.stanford.edu/public/html/) [63] were used to perform GO analysis. Briefly, gene names were first converted to DAVID-recognizable IDs using Gene Accession Conversion Tool. The updated gene list was then subjected to GO analysis using all *Mus musculus* genes or genes only expressed in mouse liver (FPKM > 0.1) as background and with the “Functional Annotation Chart” function. KEGG_PATHWAY were used as GO categories for all GO analysis. Only GO terms with *P* < 0.05 were included for further analysis. For GREAT analysis, the −500 bp to 500 bp window of TSS for each gene was input as a bed file, and enriched MSigDB pathways were generated. The criteria for associating genomic regions with genes are as follows: each gene is assigned a basal regulatory domain of a minimum distance upstream and downstream of the TSS (regardless of other nearby genes) (proximal 5 kb upstream, 1 kb downstream, plus distal up to 100 kb). The gene regulatory domain is extended in both directions to the nearest gene's basal domain but no more than the maximum extension in one direction.

### Binding site annotation and profiling

The Cis-regulatory Element Annotation System (CEAS) function in Galaxy/Cistrome (http://cistrome.org/ap/) was applied to calculate the enrichment of the binding sites in the promoter, exon, intron, UTR, and other genomic regions against the mappable mouse genome using the binomial model.

### Motif analysis

Motif analysis was performed with the Discriminative DNA Motif Discovery (DREAM) tool (version 4.9.1) or the SeqPos motif tool (version 0.590) embedded in Galaxy Cistrome using the all motifs within mouse reference genome mm10 as background. The distribution of XBP1s and GABPA motifs around XBP1s ChIP-Seq signal was generated by the CentriMo online toolbox (version 5.1.0) (http://meme-suite.org/tools/centrimo).

### Network analysis

Construction of interacting networks of evolutionarily conserved 12-h genes was performed by STRING (https://string-db.org/).

### Post hoc analysis of serum-synchronized MMH-D3 transcriptome

The time series transcriptome of serum-synchronized murine liver MMH-D3 cells was published previously [19]. Transcripts with averaged expression larger than 20 were used for subsequent analysis. Upon examining the raw data, we observed noticeable baseline changes in most reported mRNA oscillations. Therefore, we subjected the raw data to polynomial detrend (*n* = 3) (we found that higher order of polynomial detrend [*n* > 3] will lead to the disappearance of circadian rhythm in some core circadian genes, therefore we think that *n* = 3 is the optimal trade-off between overfitting and underfitting). Then, we added the mean expression of the raw data to the detrended data so that the mean expression of detrended data was equal to that of the raw data (instead of being 0). The polynomial detrended data were then subjected to eigenvalue/pencil analysis to identify superimposed oscillations, and the relative amplitude was calculated similarly as in vivo studies. Specifically, 3 oscillations were identified from each gene. The criteria for circadian genes are a period between 20.5 h and 23.5 h and a decay rate between 0.9 and 1.1; for approximately 12-h genes, the criteria are a period between 9.5 h and 12.5 h and a decay rate between 0.9 and 1.1. The smaller periods for both circadian and 12-h genes were selected based upon the distribution of all periods uncovered as shown in S3A Fig. For RAIN analysis, the detrended data underwent log 2 normalization first and then were subjected to RAIN analysis as described previously.

## Supporting information

S1 Fig Liver-specific deletion of XBP1s does not alter rhythmic locomotor activity or fasting-feeding cycles in mice, related to Fig 1. (A) Diagram showing the *Xbp1* locus of XBP1^*Flox*^ and XBP1^*LKO*^ mice (top) and RNA-Seq data depicting the absence of reads mapped to the second exon in XBP1^*LKO*^ mice (bottom). (B) Diagram showing the position of primers designed to measure the mRNA level of *Xbp1us* and *Xbp1s*. (C) Western blot analysis of total hepatic XBP1s in XBP1^*Flox*^ and XBP1^*LKO*^ mice. (D, E) Real-time home cage activity monitoring of total distance covered (top), number of movements recorded (middle), and movement time recorded (bottom) in XBP1^*Flox*^ and XBP1^*LKO*^ mice under 12-h light/12-h dark conditions (panel D) and constant darkness condition (panel E). (F) Averaged measurements within the first and second 12 h of a day as described in panels D and E. (G) Real-time measurement of food intake in XBP1^*Flox*^ and XBP1^*LKO*^ mice under both 12-h light/12-h dark and constant-darkness condition measured by the CLAMS system. (H) Averaged measurements within the first and second 12 h of a day as described in panel D. Data are graphed as the mean ± SEM (*n* = 3–4). Numerical values are available in S5 Data.
(TIF)

S2 Fig Liver-specific deletion of XBP1s impairs global hepatic 12-h transcriptome, but not the circadian rhythm, in mice, related to Figs 1 and 2. (A) Permutation was performed on the raw data by randomly shuffling the time label. Distribution of periods of all oscillations identified by the eigenvalue/pencil method from 4 representative permutated datasets from XBP1^*Flox*^ mice. (B, C) UCSC genome browser snapshot view of RNA-Seq tracks of selective circadian (panel B) and 12-h cycling (panel C) gene expression in XBP1^*Flox*^ mice and XBP1^*LKO*^ mice. (D) Genes with superimposed 24-h rhythms found in both XBP1^*Flox*^ and XBP1^*LKO*^ mice. GO analysis showing enriched KEGG pathways and their corresponding *P* values (top) and RNA-Seq data for representative genes (bottom). (E) Genes with superimposed 24-h rhythms only found in XBP1^*Flox*^ mice. GO analysis showing enriched KEGG pathways and their corresponding *P* values (top) and RNA-Seq data for representative genes (bottom). (F, G) GO analysis of all XBP1s-dependent 12-h genes showing enriched KEGG pathways using either all mouse genes (panel F) or hepatic expressed genes (panel G) as background, with corresponding *P* values ranked. GO associated with CEDIF are highlighted in red. (H, I) GO analysis of all XBP1s-dependent 12-h genes showing enriched GOTERM_BP_DIRECT pathways using either all mouse genes (panel H) or hepatic expressed genes (panel I) as background, with corresponding *P* values ranked. GO associated with CEDIF are highlighted in red. Numerical values are available in S5 Data.
(TIF)

S3 Fig Prevalent XBP1s-dependent 12-h hepatic transcriptome revealed by RAIN, related to Fig 3. (A) Heat map of the expression of 12-h cycling genes identified by RAIN, with FDR < 0.001 and FDR < 0.01. Heat map showing the log_10_ transformed Benjamini-Hochberg procedure–adjusted *P* value for each identified 12-h gene was shown on the right. (B) Heat map of the expression of 12-h cycling genes identified in both XBP1^*Flox*^ and XBP1^*LKO*^ mice by RAIN with 3 different FDR cut-offs ranked according to the phase in XBP1^*LKO*^ mice. (C) Venn diagram comparison of 12-h transcriptome uncovered by the eigenvalue and RAIN methods (with different FDR cut-offs of 0.001, 0.01, and 0.05) in XBP1^*Flox*^ mice. (D) Distribution of the periods of dominant oscillations uncovered by the eigenvalue method for the 1,288 genes whose 12-h rhythms are specifically identified by the RAIN method in XBP1^*Flox*^ mice. (E) RNA-Seq data for 2 representative genes selected from these 1,288 genes in both XBP1^*Flox*^ and XBP1^*LKO*^ mice. The period (red) is calculated by the eigenvalue method for the dominant oscillation in each gene in XBP1^*Flox*^ mice. The two *P* values (indicating how robust their 12-h rhythms are) are calculated by the RAIN method for each gene in XBP1^*Flox*^ (black) and XBP1^*LKO*^ (cyan) mice, respectively. (F) Distribution of the periods of dominant oscillations uncovered by the eigenvalue method for the 2,009 genes whose 12-h rhythms are specifically identified by the eigenvalue method in XBP1^*Flox*^ mice. (G) RNA-Seq data for 2 representative genes selected from these 2,009 genes in XBP1^*Flox*^ mice. Both the original (black) and circadian rhythm removed (green) expressions are shown. The period (red) is calculated by the eigenvalue method for the superimposed 12-h oscillation present in each gene in XBP1^*Flox*^ mice. The two *P* values (indicating how robust their 12-h rhythms are) are calculated by the RAIN method for each gene in the original (black) and circadian-rhythm-removed (green) data, respectively. (H) Distribution of the periods of dominant oscillations uncovered by the eigenvalue method for the 2,590 genes whose 12-h rhythms are identified by both methods in XBP1^*Flox*^ mice. (I) RNA-Seq data for 2 representative genes selected from these 2,590 genes in both XBP1^*Flox*^ and XBP1^*LKO*^ mice. The period (red) is calculated by the eigenvalue method for the 12-h oscillation in each gene in XBP1^*Flox*^ mice. The two *P* values (indicating how robust their 12-h rhythms are) are calculated by the RAIN method for each gene in XBP1^*Flox*^ (black) and XBP1^*LKO*^ (cyan) mice, respectively. (J) Venn diagram comparison of 12-h transcriptome uncovered by the eigenvalue, RAIN method (with the FDR cut-off of 0.05), RAIN method (with the FDR cut-off of 0.05) using circadian rhythm removed data in XBP1^*Flox*^ mice. (K) Venn diagram comparison of XBP1s-dependent 12-h transcriptome uncovered by the eigenvalue and RAIN + DODR methods (FDR < 0.05), with a short list of representative commonly and uniquely identified genes. (L–M) RNA-Seq data for cell cycle gene *Cdk2* (panel L) and MAPK gene *Dusp10* (panel M) in XBP1^*Flox*^ and XBP1^*LKO*^ mice, as well as data fit (dashed red line) by adding up all 3 superimposed oscillations uncovered by the eigenvalue method in XBP1^*Flox*^ mice (left). Three superimposed oscillations identified by the eigenvalue method in XBP1^*Flox*^ mice (right). (N, O) RNA-Seq data for *Sec61a1* (panel N) and *Ddx55* (panel O) in XBP1^*Flox*^ and XBP1^*LKO*^ mice as well as DODR *P* value and FDR cut-off. Numerical values are available in S5 Data.
(TIF)

S4 Fig Nondominant 12-h rhythm genes are also under XBP1s-dependent 12-h clock control, related to Fig 3. (A, B) Venn diagram showing the common and unique circadian and 12-h transcriptome identified by the RAIN method (FDR < 0.05) (A) or the eigenvalue method (B) in XBP1^*Flox*^ mice. (C) Venn diagram showing the common and unique 12-h transcriptome identified by the eigenvalue method (both total and dominant) and by the RAIN method in XBP1^*Flox*^ mice. (D, G) KEGG enriched pathways of 1,428 (D) or 1,458 (G) “non-dominant” 12-h transcriptome. (E, H) Representative RNA-Seq and eigenvalue decomposition of *Sec63* (panel E) or *Sedt1b* (panel H). (F, I) Heat map of commonly found and XBP1^*Flox*^ mice-specific 12-h transcriptome for the 1,428 (panel F) and 1,458 (panel I) genes. Numerical values are available in S5 Data.
(TIF)

S5 Fig The 12-h rhythms of CEDIF gene expression are cell autonomous, related to Fig 4. (A, B) Distribution of periods of all (A) and dominant oscillations (B) identified by eigenvalue/pencil method from MMH-D3 cells. (C, D) Heat map of all circadian (C) and core circadian clock (D) gene expression identified from MMH-D3 cells with both raw data and superimposed 24-h rhythms shown. Both the original time after serum shock as well as converted time in CT are shown. (E) GO analysis showing enriched KEGG pathways and their corresponding *P* values for all circadian gene identified in panel C. (F) Venn diagram comparison of all 12-h transcriptome from mouse liver in vivo and MMH-D3 cells in vitro. (G, H) GO analysis showing enriched KEGG pathways and their corresponding *P* values for all 1,529 commonly found 12-h transcriptomes using all mouse genes (G) or all hepatic expressed genes (H) as background. (I) RNA-Seq (top) and microarray (bottom) data of representative 12-h immune genes in mouse liver (top) and MMH-D3 cells (bottom). (J) Microarray data of representative 12-h cycling genes involved in CEDIF in MMH-D3 cells that are commonly shared with mouse liver. *P* values indicating the robustness of 12-h rhythm detection by RAIN are also shown for each gene. (K) Microarray data of representative 12-h cycling genes involved in CEDIF uniquely found in MMH-D3 cells. *P* values indicating the robustness of 12-h rhythm detection by RAIN are also shown for each gene. (L) Polar histograms demonstrating phase distributions of 12-h genes involved in different steps of CEDIF in MMH-D3 cells. (M) MEFs were transfected with different siRNAs and treated with dexamethasone (100 nM) for 30 min. Western blot analysis of XBP1s and BMAL1 (top left), and qPCR was performed at different time points post dexamethasone shock. (N) Real-time luminescence analysis of *Bmal1-dluc* MEFs post 50% horse serum shock. Representative raw and detrended traces of luminescence recordings from MEFs subjected to different siRNA transfection (top) and quantified amplitude, period, and phases (bottom). (O) Distribution of periods of all oscillating metabolites identified by the eigenvalue/pencil method compiled from a published metabolome dataset in human U2OS cells [24] (top) and calculated periods of approximately 12-h metabolites (bottom) in the presence of different siRNAs. Data are graphed as the mean ± SEM (*n* = 2–9). Numerical values are available in S5 Data.
(TIF)

S6 Fig The motif stringency of XBP1s promoter binding sites dictates XBP1s’s ability to drive 12-h rhythms of nascent mRNA transcription, related to Figs 5 and 6. (A) Venn diagram depicting common and unique XBP1s cistrome and those of core circadian clock transcription factors compiled from [40,57,64]. (B) Snapshot of selected genes for alignment of hepatic XBP1s binding sites at different CTs in XBP1^*Flox*^ and XBP1^*LKO*^ mice. (C) Snapshot of selected genes for alignment of hepatic XBP1s binding sites at bidirectional promoters at different CTs in XBP1^*Flox*^ and XBP1^*LKO*^ mice. (D) Percentage of XBP1s cistromes identified at different positions relative to target genes compared with that of mouse genome. (E) RNA-Seq data for *Creb3l2*, *Elf1*, and *Atf6* in XBP1^*Flox*^ and XBP1^*LKO*^ mice. (F) Western blot analysis and quantification of temporal ATF6 and XBP1s levels in mouse liver. (G) RNA-Seq data for *Gabpa* and *Gabpb1* in XBP1^*Flox*^ and XBP1^*LKO*^ mice with calculated periods in XBP1^*Flox*^ mice shown (panel F). (H) Snapshot of *Gabpa* promoter for alignment of hepatic XBP1s binding sites at different CTs in XBP1^*Flox*^ and XBP1^*LKO*^ mice. (I) Nuclear level of GABPA and nuclear level of GABPA and GABPB2 bound to DNA compiled from [38]. (J) GO analysis showing enriched MSiIgDB pathways and their corresponding *P* values for 3,730 12-h intron-mapping transcriptome without XBP1s binding sites. (K) Top enriched SeqPos motifs common to proximal promoters (1,000 bp around TSS) of 3,730 twelve-hour intron-mapping transcriptome without XBP1s binding sites. (L) Polar histogram demonstrating phase distributions of 3,730 intron-mapping 12-h cycling genes without XBP1s binding in XBP1^*Flox*^ mice. (M) Log_2_ mean-normalized transcription elongation rates calculated from the Gro-Seq data [42] for XBP1s target genes with or without associated 12-h transcriptome. (N) Snapshot of target genes selected for alignment of hepatic XBP1s binding sites at different CTs in XBP1^*Flox*^ and XBP1^*LKO*^ mice as well as published Gro-Seq data [42]. Consensus XBP1s binding motifs identified at each gene promoter are also shown; (O–R) 699 genes with proximal promoter XBP1s binding but without 12-h intron-mapping transcriptome in XBP1^*Flox*^ mice. (O) Comparisons of heat maps of XBP1s binding intensity, transcription initiation rates calculated from Gro-Seq [42], intron-mapping gene expression in XBP1^*Flox*^ and XBP1^*LKO*^ mice, and XBP1s binding motif score. (P) Average expression for each gene in XBP1^*Flox*^ and XBP1^*LKO*^ mice. (Q) Snapshot of *Aasdh* gene selected for alignment of hepatic XBP1s binding sites at different CTs in XBP1^*Flox*^ and XBP1^*LKO*^ mice as well as published Gro-Seq data [42]. Degenerate XBP1s binding motifs identified at gene promoter are also shown. (R) RNA-Seq data (both intron and exon mapping) for *Aasdh* gene in XBP1^*Flox*^ and XBP1^*LKO*^ mice. Data are graphed as mean ± SEM (*n* = 2). Numerical values are available in S5 Data except for S6D Fig, which was automatically generated by the CEAS toolbox in Galaxy/Cistrome.
(TIF)

S7 Fig The 12-h rhythms of CEDIF gene expression are evolutionarily conserved in the limpet *C. rota*, which possesses a circatidal clock, related to Fig 7. (A) Heat map of side-by-side comparison of evolutionarily conserved 12-h gene expression in *C*. *rota* [10] and mouse liver, with both raw data and superimposed circatidal rhythms shown. The level of tides corresponding to each time point is also shown. (B) Predicted interactive network construction of these conserved 12-h cycling genes using STRING [49]. Genes involved in different biological pathways are colored differently. (C) RNA-Seq data for representative genes in *C*. *rota* [10] and in XBP1^*Flox*^ and XBP1^*LKO*^ mice. (D) RNA-Seq data for *Gabpa* in *C*. *rota*. Data are graphed as the mean ± SEM (*n* = 2). Numerical values are available in S5 Data. A higher-resolution image in panel B is available in S3 Raw images.
(TIF)

S1 Table Alignment statistics for RNA-Seq, related to Fig 1. The number of total and mapped reads for RNA-Seq data.
(XLSX)

S2 Table FPKM quantification of RNA-Seq data in XBP1*^Flox^* and XBP1*^LKO^* mice, related to Fig 1. Gene ID, coordinate (mm10), and FPKM quantification at each CT in XBP1^*Flox*^ and XBP1^*LKO*^ mice are shown.
(RAR)

S3 Table Eigenvalue/pencil decomposition of all transcriptomes with means larger than 0.1 in XBP1*^Flox^* and XBP1*^LKO^* mice, related to Fig 1. Period, decay rate, biological phase (time of peak), and amplitude for each gene are provided as well as the mean value. Relative amplitude was calculated by normalizing the absolute amplitude to the mean. Tab 1: Basic statistics of distribution of period and decay rates for all oscillations. Tab 2: Genes in XBP1^*Flox*^ mice. Tab 3: Genes in XBP1^*LKO*^ mice.
(XLSX)

S4 Table GO terms associated with genes with 12-h rhythms of expression, related to Fig 2. Tab 1: GO terms associated with all genes with 12-h rhythms in XBP1^*Flox*^ mice, identified using the eigenvalue/pencil method, using all mouse genes as background. Tab 2: GO terms associated with all genes with 12-h rhythms in XBP1^*Flox*^ mice, identified using the eigenvalue/pencil method, using all expressed mouse genes as background. Tab 3: GO terms associated with the genes whose 12-h rhythms were either abolished or dampened in XBP1^*LKO*^ compared to XBP1^*Flox*^ mice, identified using the eigenvalue/pencil method, using all mouse genes as background. Tab 4: GO terms associated with the genes whose 12-h rhythms were either abolished or dampened in XBP1^*LKO*^ compared to XBP1^*Flox*^ mice, identified using the eigenvalue/pencil method, using all expressed mouse genes as background. Tab 5: GO terms associated with all 2,501 genes with 12-h rhythms only in XBP1^*Flox*^ mice, identified using the eigenvalue/pencil method, using all 12-h mouse genes as background. Tab 6: GO terms associated with all 1,454 genes with 12-h rhythms larger in XBP1^*Flox*^ mice, identified using the eigenvalue/pencil method, using all 12-h mouse genes as background. Tab 7: GO terms associated with all 638 genes with 12-h rhythms smaller in XBP1^*Flox*^ mice, identified using the eigenvalue/pencil method, using all 12-h mouse genes as background. Tab 8: GO terms associated with commonly found 12-h rhythms in XBP1^*Flox*^ mice, identified using the eigenvalue/pencil and RAIN methods (FDR < 0.05), using all mouse genes as background. Tab 9: GO terms associated with commonly found 12-h rhythms in XBP1^*Flox*^ mice, identified using the eigenvalue/pencil and RAIN methods (FDR < 0.05), using all expressed mouse genes as background. Tab 10: GO terms associated with all genes with 12-h rhythms in XBP1^*Flox*^ mice, identified using the RAIN method (FDR < 0.05), using all mouse genes as background. Tab 11: GO terms associated with all genes with 12-h rhythms in XBP1^*Flox*^ mice, identified using the RAIN method (FDR < 0.05), using all expressed mouse genes as background. Tab 12: GO terms associated with all genes with 12-h rhythms in XBP1^*Flox*^ mice, identified using the RAIN method (FDR < 0.01), using all mouse genes as background. Tab 13: GO terms associated with all genes with 12-h rhythms in XBP1^*Flox*^ mice, identified using the RAIN method (FDR < 0.01), using all expressed mouse genes as background. Tab 14: GO terms associated with all genes with 12-h rhythms in XBP1^*Flox*^ mice, identified using the RAIN method (FDR < 0.001), using all mouse genes as background. Tab 15: GO terms associated with all genes with 12-h rhythms in XBP1^*Flox*^ mice, identified using the RAIN method (FDR < 0.001), using all expressed mouse genes as background. Tab 16: GO terms associated with all 12-h genes abolished or with amplitude dampened in XBP1^*LKO*^ mice using the RAIN and DODR method (FDR < 0.05), using all mouse genes as background. Tab 17: GO terms associated with all 12-h genes abolished or with amplitude dampened in XBP1^*LKO*^ mice using the RAIN and DODR method (FDR < 0.05), using expressed mouse genes as background.
(XLSX)

S5 Table Lists of genes involved in transcription, RNA metabolism, protein metabolism in the ER, maintaining Golgi integrity and function, whose 12-h rhythms were either abolished or dampened in XBP1*^LKO^* compared to XBP1*^Flox^* mice, related to Fig 2. Twelve-hour period, decay rate, biological phase, and amplitude for each gene were provided as well as the mean value. Relative amplitude was calculated by normalizing the absolute amplitude to the mean. If a 12-h rhythm is not found, it is indicated by “N/A.” Tab 1: Genes involved in transcription. Tab 2: Genes involved in RNA metabolism. Tab 3: Genes involved in ribosome biogenesis. Tab 4: Genes involved in protein metabolism. Tab 5: Genes involved in maintaining Golgi integrity and function.
(XLSX)

S6 Table Analysis of hepatic transcriptome by RAIN, related to Fig 3. Tab 1: 12-h rhythm identified by RAIN in XBP1^*Flox*^ mice. Tab 2: 12-h rhythm identified by RAIN in XBP1^*LKO*^ mice. Tab 3: 24-h rhythm identified by RAIN in XBP1^*Flox*^ mice. Tab 4: 24-h rhythm identified by RAIN in XBP1^*LKO*^ mice. Tab 5: 12-h rhythm identified by RAIN in XBP1^*Flox*^ mice, all superimposed 24-h removed.
(XLSX)

S7 Table DODR analysis of commonly found 12-h genes by RAIN (FDR < 0.05), related to Fig 4. Tab 1: DODR analysis demonstrating differentially expressed 12-h genes that have a lower amplitude in XBP1 ^*LKO*^ mice. Tab 2: DODR analysis demonstrating differentially expressed 12-h genes that have a higher amplitude in XBP1 ^*LKO*^ mice. Tab 3: the list of 1,573 XBP1s-dependent 12-h cycling hepatic genes that are commonly identified by both eigenvalue and RAIN + DODR methods (FDR < 0.05).
(XLSX)

S8 Table Eigenvalue/pencil decomposition of MMH-D3 transcriptome, related to Fig 4. Period, decay rate, biological phase, and amplitude for each gene were provided as well as the mean value. Relative amplitude was calculated by normalizing the absolute amplitude to the mean. Tab 1: all genes. Tab 2: 12-h genes. Tab 3: Circadian genes.
(XLSX)

S9 Table Analysis of MMH-D3 transcriptome by RAIN, related to Fig 4. Tab 1: 12-h rhythm identified by RAIN. Tab 2: A list of 12-h genes that are shared between the eigenvalue/pencil and RAIN methods.
(XLSX)

S10 Table List of 12-h cycling CEDIF genes uniquely and commonly found in mouse liver in vivo and MMH-D3 cells in vitro, related to Fig 4. (XLSX)

S11 Table GO terms associated with 12-h cycling genes in MMH-D3 cells, related to Fig 4. (XLSX)

S12 Table Quantification and eigenvalue/pencil decomposition of XBP1s ChIP-Seq, related to Fig 5. RPKM quantification and eigenvalue/pencil-identified 12-h rhythm of XBP1s cistrome.
(XLSX)

S13 Table FPKM quantification of intron-mapping RNA-Seq data in XBP1^*Flox*^ and XBP1^*LKO*^ mice, related to Fig 6. Gene ID, coordinate (mm10), and FPKM quantification at each CT in XBP1^*Flox*^ and XBP1^*LKO*^ mice are shown.
(RAR)

S14 Table GO terms associated with all XBP1s-bound genes, related to Fig 6. (XLSX)

S15 Table Eigenvalue results of circatidal transcripts in *C. rota* and list of genes with or without conserved 12-h rhythms in *C. rota* or *A. diaphaha*, related to Fig 7 and S7 Fig. Tab 1: eigenvalue results of circatidal genes in *C*. *rota*. Circatidal period, decay rate, mathematical and biological phase, and amplitude for each gene were provided as well as the mean value. Relative amplitude was calculated by normalizing the absolute amplitude to the mean. Tab 2: gene list of unique and evolutionarily conserved (in mouse) circatidal genes in *C*. *rota* and *A*. *diaphaha*.
(XLSX)

S16 Table GO terms associated with all and conserved 12-h genes in *C. rota* and *A. diaphaha*, related to Fig 7 and S7 Fig. Tab 1: All circatidal genes in *C*. *rota*. Tab 2: All conserved 12-h genes in mouse and *C*. *rota*. Tab 3: All circatidal genes in *A*. *diaphaha*. Tab 4: All conserved 12-h genes in mouse and *A*. *diaphaha*.
(XLSX)

S1 Raw images Original images of western blot gels shown in S1C Fig, S5M Fig and S6F Fig. (PDF)

S2 Raw image High-resolution image of Fig 7B. (PDF)

S3 Raw image High-resolution image of S7B Fig. (PDF)

S1 Data Original numerical values for Fig 1A, 1B, 1D, 1E, 1F, 1G, 1H, 1I and 1J. (XLSX)

S2 Data Original numerical values for Figs 1K, 2B, 2C, 2E and 2F. (XLSX)

S3 Data Original numerical values for Fig 3A, 3B, 3D, 3E, 3F and 3H. (XLSX)

S4 Data Original numerical values for Figs 4A, 4D, 4E, 4F, 4G, 4H, 4I, 4J, 4K, 5C, 5F, 5G, 6D, 6E, 6G, 6H, 6I, 6J, 6K, 6M, 7A, 7C and 7D. (XLSX)

S5 Data Original numerical values for S1D, S1E, S1F, S1G, S1H, S2A, S2D, S2E, S2F, S2G, S2H, S2I, S3D, S3E, S3F, S3G, S3H, S3I, S3L, S3M, S3N, S3O, S4E, S4F, S4H, S4I, S5A, S5B, S5C, S5D, S5I, S5J, S5K, S5L, S5M, S5N, S5O, S6C, S6E, S6F, S6G, S6I, S6L, S6M, S6N, S6P, S6R, S7A, S7C and S7D Figs. (XLSX)

## Abbreviations

ALG: Asparagine-Linked Glycosylation
ATF6: Activating Transcription Factor 6
BSA: bovine serum albumin
CCR4-NOT: Carbon Catabolite Repressor 4-Negative on TATA
CEAS: Cis-regulatory Element Annotation System
CEDIF: central dogma information flow
ChIP-Seq: chromatin immunoprecipitation sequencing
CLAMS: Comprehensive Lab Animal Monitoring System
COPII: Coatomer II
Cpsf: cleavage and polyadenylation specificity factor
CREB3: Cyclic AMP-Responsive Element-Binding protein 3
CREB3L: Cyclic AMP-Responsive Element-Binding protein 3-Like
CT: constant time
Ctsf: cleavage stimulatory factor
dGFP: destabilized green fluorescent protein
DODR: Detection of Differential Rhythmicity
DREAM: Discriminative DNA Motif Discovery
ELF1: E74 Like ETS Transcription Factor 1
ER: Endoplasmic Reticulum
ETS: E26 Transformation-Specific
FDR: false discovery rate
FPKM: Fragments Per Kilobase of transcript per Million mapped reads
GABP: GA-binding protein
GO: Gene Ontology
Gro-Seq: Global run-on Sequencing
HIF-1: Hypoxia-Inducible Factors
HOV: high-occupancy vehicle
HSP40: Heat Shock Protein 40 kD
KEGG: Kyoto Encyclopedia of Genes and Genomes
MAPK: Mitogen-Activated Protein Kinase
MEF: mouse embryonic fibroblast
MHC: major histocompatibility complex
MSigDB: Molecular Signatures Database
PERK: Protein kinase R-like Endoplasmic Reticulum Kinase
qPCR: quantitative PCR
RAIN: Rhythmicity Analysis Incorporating Nonparametric
RNA-Seq: RNA sequencing
SeqPos: Sequence Position
siRNA: small interfering RNA
SRP: Signal Recognition Particle
STRING: Search Tool for the Retrieval of Interacting Genes/Proteins
TES: transcription termination site
TNF: tumor necrosis factor
t-SNE: t-distributed Stochastic Neighbor Embedding
TSS: transcription start site
UPR: unfolded protein response
XBP1s: Spliced Form of X-box Binding Protein 1
XBP1us: unspliced form of XBP1
ZT: Zeitgeber time
