# Supplementary figures and images for: 12-h clock regulation of genetic information flow by XBP1s
Source: PLoS Biol. 2020 Jan 14;18(1):e3000580. doi: 10.1371/journal.pbio.3000580 (PMC6959563; doi:10.1371/journal.pbio.3000580)

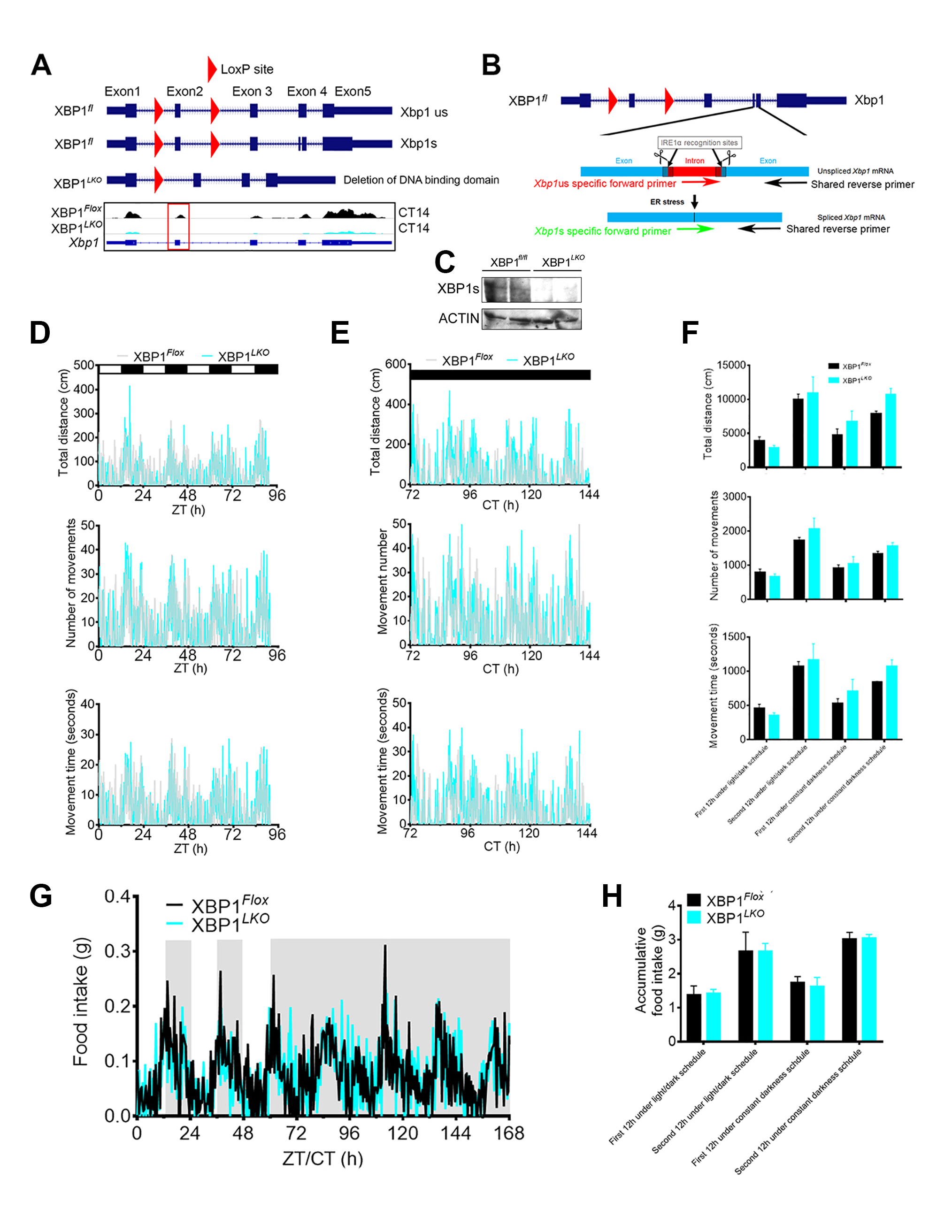

Supplement: S1 Fig — (A) Diagram showing the Xbp1 locus of XBP1Flox and XBP1LKO mice (top) and RNA-Seq data depicting the absence of reads mapped to the second exon in XBP1LKO mice (bottom). (B) Diagram showing the position of primers designed to measure the mRNA level of Xbp1us and Xbp1s. (C) Western blot analysis of total hepatic XBP1s in XBP1Flox and XBP1LKO mice. (D, E) Real-time home cage activity monitoring of total distance covered (top), number of movements recorded (middle), and movement time recorded (bottom) in XBP1Flox and XBP1LKO mice under 12-h light/12-h dark conditions (panel D) and constant darkness condition (panel E). (F) Averaged measurements within the first and second 12 h of a day as described in panels D and E. (G) Real-time measurement of food intake in XBP1Flox and XBP1LKO mice under both 12-h light/12-h dark and constant-darkness condition measured by the CLAMS system. (H) Averaged measurements within the first and second 12 h of a day as described in panel D. Data are graphed as the mean ± SEM (n = 3–4). Numerical values are available in S5 Data. (TIF) [file pbio.3000580.s001.tif]

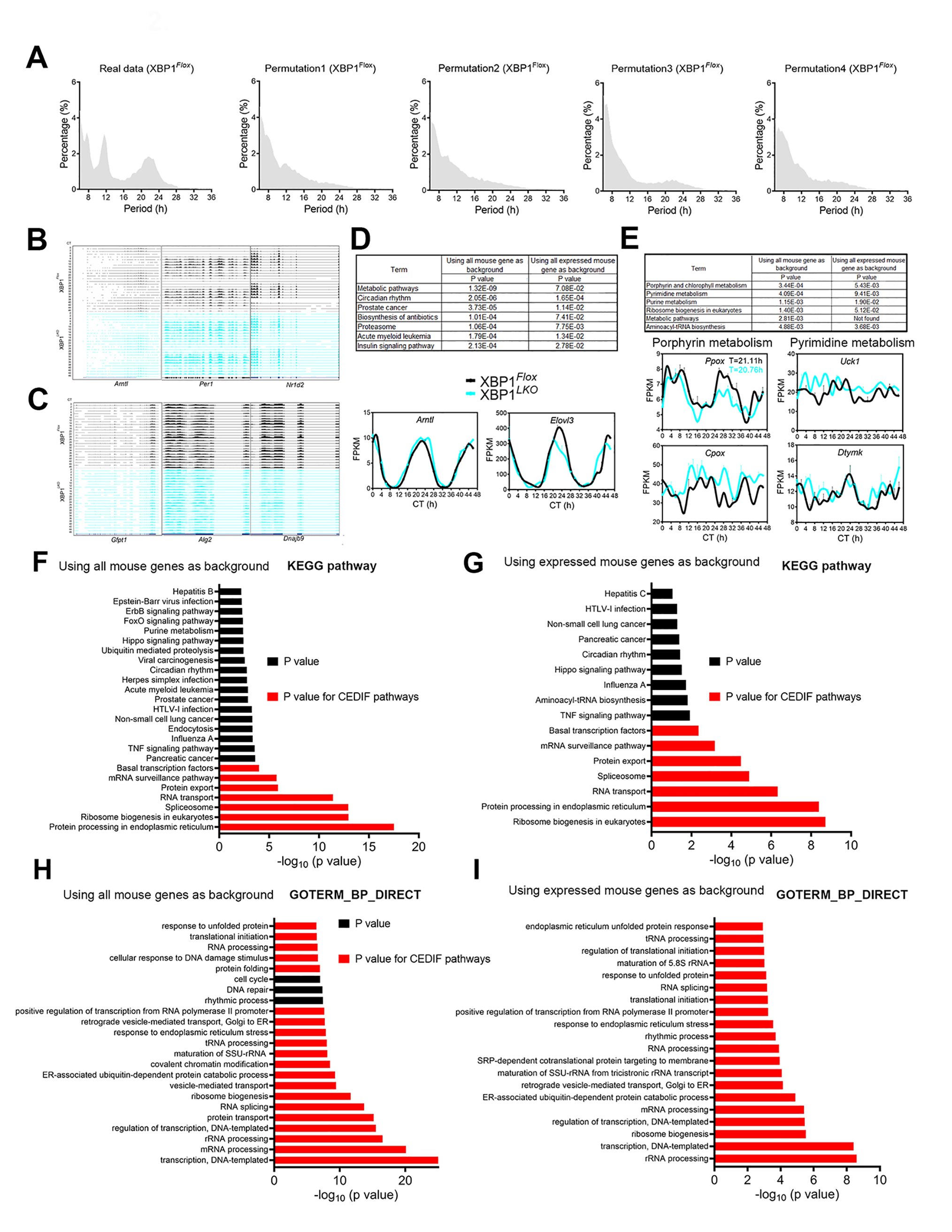

Supplement: S2 Fig — (A) Permutation was performed on the raw data by randomly shuffling the time label. Distribution of periods of all oscillations identified by the eigenvalue/pencil method from 4 representative permutated datasets from XBP1Flox mice. (B, C) UCSC genome browser snapshot view of RNA-Seq tracks of selective circadian (panel B) and 12-h cycling (panel C) gene expression in XBP1Flox mice and XBP1LKO mice. (D) Genes with superimposed 24-h rhythms found in both XBP1Flox and XBP1LKO mice. GO analysis showing enriched KEGG pathways and their corresponding P values (top) and RNA-Seq data for representative genes (bottom). (E) Genes with superimposed 24-h rhythms only found in XBP1Flox mice. GO analysis showing enriched KEGG pathways and their corresponding P values (top) and RNA-Seq data for representative genes (bottom). (F, G) GO analysis of all XBP1s-dependent 12-h genes showing enriched KEGG pathways using either all mouse genes (panel F) or hepatic expressed genes (panel G) as background, with corresponding P values ranked. GO associated with CEDIF are highlighted in red. (H, I) GO analysis of all XBP1s-dependent 12-h genes showing enriched GOTERM_BP_DIRECT pathways using either all mouse genes (panel H) or hepatic expressed genes (panel I) as background, with corresponding P values ranked. GO associated with CEDIF are highlighted in red. Numerical values are available in S5 Data. (TIF) [file pbio.3000580.s002.tif]

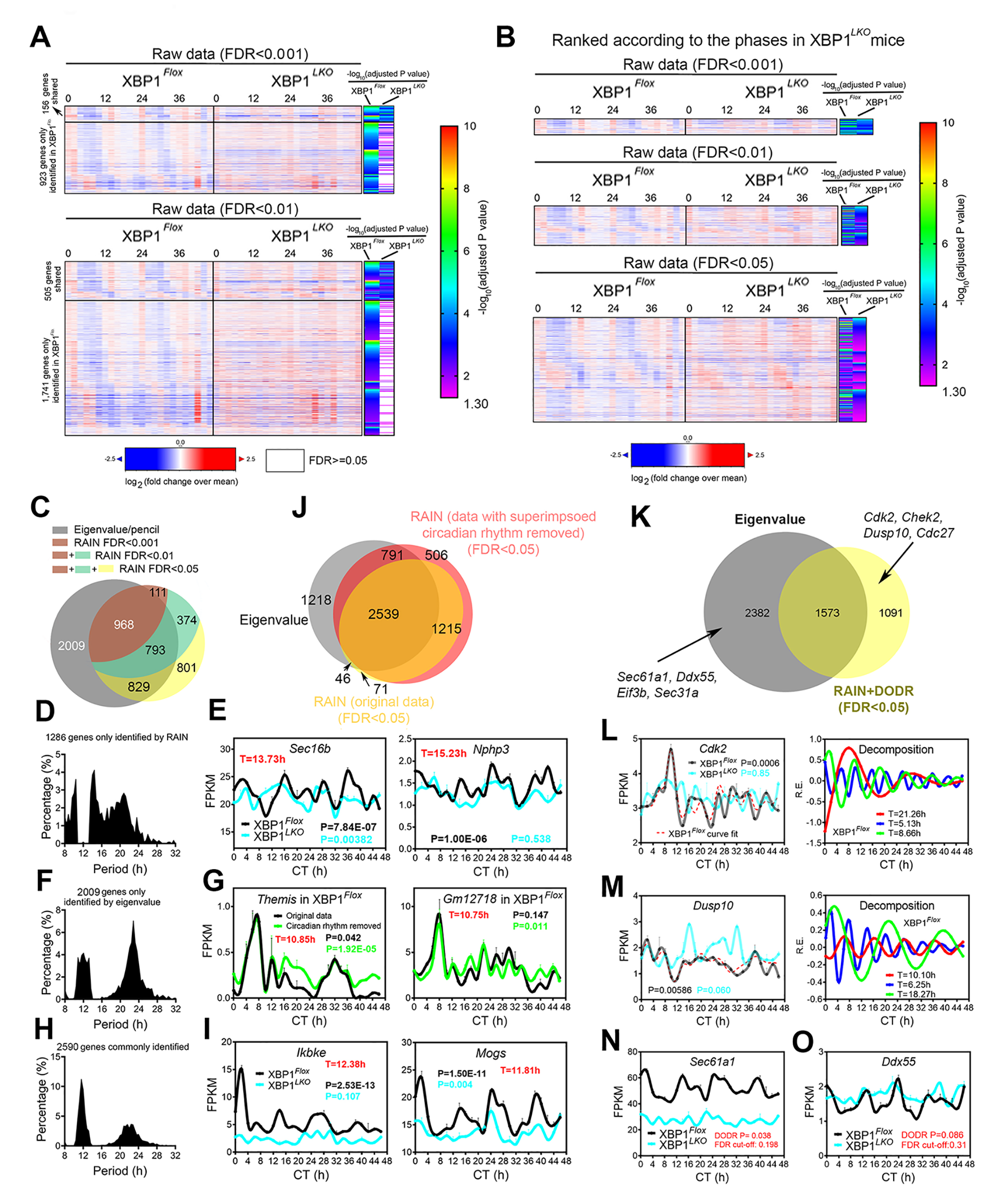

Supplement: S3 Fig — (A) Heat map of the expression of 12-h cycling genes identified by RAIN, with FDR < 0.001 and FDR < 0.01. Heat map showing the log10 transformed Benjamini-Hochberg procedure–adjusted P value for each identified 12-h gene was shown on the right. (B) Heat map of the expression of 12-h cycling genes identified in both XBP1Flox and XBP1LKO mice by RAIN with 3 different FDR cut-offs ranked according to the phase in XBP1LKO mice. (C) Venn diagram comparison of 12-h transcriptome uncovered by the eigenvalue and RAIN methods (with different FDR cut-offs of 0.001, 0.01, and 0.05) in XBP1Flox mice. (D) Distribution of the periods of dominant oscillations uncovered by the eigenvalue method for the 1,288 genes whose 12-h rhythms are specifically identified by the RAIN method in XBP1Flox mice. (E) RNA-Seq data for 2 representative genes selected from these 1,288 genes in both XBP1Flox and XBP1LKO mice. The period (red) is calculated by the eigenvalue method for the dominant oscillation in each gene in XBP1Flox mice. The two P values (indicating how robust their 12-h rhythms are) are calculated by the RAIN method for each gene in XBP1Flox (black) and XBP1LKO (cyan) mice, respectively. (F) Distribution of the periods of dominant oscillations uncovered by the eigenvalue method for the 2,009 genes whose 12-h rhythms are specifically identified by the eigenvalue method in XBP1Flox mice. (G) RNA-Seq data for 2 representative genes selected from these 2,009 genes in XBP1Flox mice. Both the original (black) and circadian rhythm removed (green) expressions are shown. The period (red) is calculated by the eigenvalue method for the superimposed 12-h oscillation present in each gene in XBP1Flox mice. The two P values (indicating how robust their 12-h rhythms are) are calculated by the RAIN method for each gene in the original (black) and circadian-rhythm-removed (green) data, respectively. (H) Distribution of the periods of dominant oscillations uncovered by the eigenvalue method for the 2, [file pbio.3000580.s003.tif]

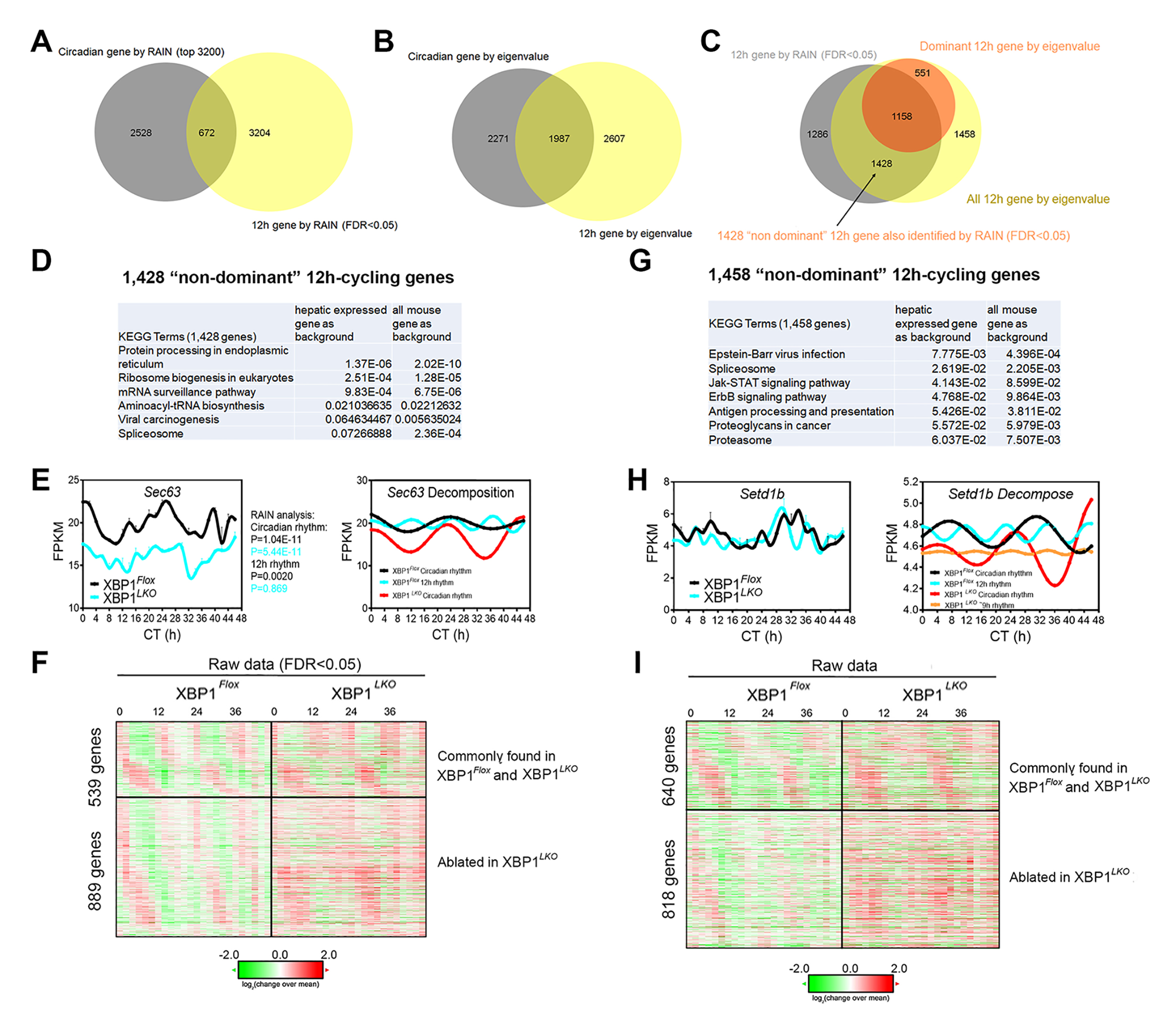

Supplement: S4 Fig — (A, B) Venn diagram showing the common and unique circadian and 12-h transcriptome identified by the RAIN method (FDR < 0.05) (A) or the eigenvalue method (B) in XBP1Flox mice. (C) Venn diagram showing the common and unique 12-h transcriptome identified by the eigenvalue method (both total and dominant) and by the RAIN method in XBP1Flox mice. (D, G) KEGG enriched pathways of 1,428 (D) or 1,458 (G) “non-dominant” 12-h transcriptome. (E, H) Representative RNA-Seq and eigenvalue decomposition of Sec63 (panel E) or Sedt1b (panel H). (F, I) Heat map of commonly found and XBP1Flox mice-specific 12-h transcriptome for the 1,428 (panel F) and 1,458 (panel I) genes. Numerical values are available in S5 Data. (TIF) [file pbio.3000580.s004.tif]

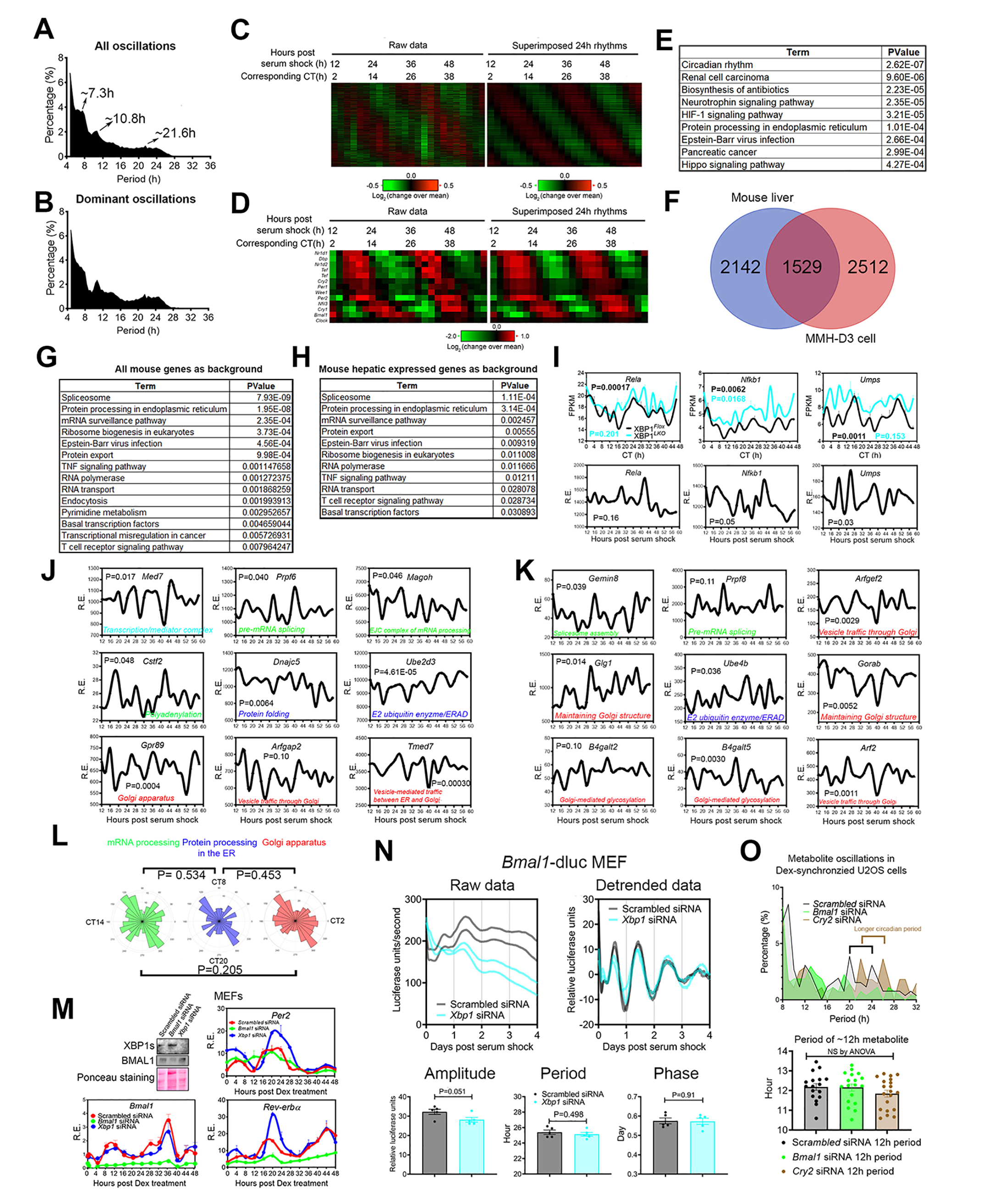

Supplement: S5 Fig — (A, B) Distribution of periods of all (A) and dominant oscillations (B) identified by eigenvalue/pencil method from MMH-D3 cells. (C, D) Heat map of all circadian (C) and core circadian clock (D) gene expression identified from MMH-D3 cells with both raw data and superimposed 24-h rhythms shown. Both the original time after serum shock as well as converted time in CT are shown. (E) GO analysis showing enriched KEGG pathways and their corresponding P values for all circadian gene identified in panel C. (F) Venn diagram comparison of all 12-h transcriptome from mouse liver in vivo and MMH-D3 cells in vitro. (G, H) GO analysis showing enriched KEGG pathways and their corresponding P values for all 1,529 commonly found 12-h transcriptomes using all mouse genes (G) or all hepatic expressed genes (H) as background. (I) RNA-Seq (top) and microarray (bottom) data of representative 12-h immune genes in mouse liver (top) and MMH-D3 cells (bottom). (J) Microarray data of representative 12-h cycling genes involved in CEDIF in MMH-D3 cells that are commonly shared with mouse liver. P values indicating the robustness of 12-h rhythm detection by RAIN are also shown for each gene. (K) Microarray data of representative 12-h cycling genes involved in CEDIF uniquely found in MMH-D3 cells. P values indicating the robustness of 12-h rhythm detection by RAIN are also shown for each gene. (L) Polar histograms demonstrating phase distributions of 12-h genes involved in different steps of CEDIF in MMH-D3 cells. (M) MEFs were transfected with different siRNAs and treated with dexamethasone (100 nM) for 30 min. Western blot analysis of XBP1s and BMAL1 (top left), and qPCR was performed at different time points post dexamethasone shock. (N) Real-time luminescence analysis of Bmal1-dluc MEFs post 50% horse serum shock. Representative raw and detrended traces of luminescence recordings from MEFs subjected to different siRNA transfection (top) and quantified amplitude, period, and phases (bottom) [file pbio.3000580.s005.tif]

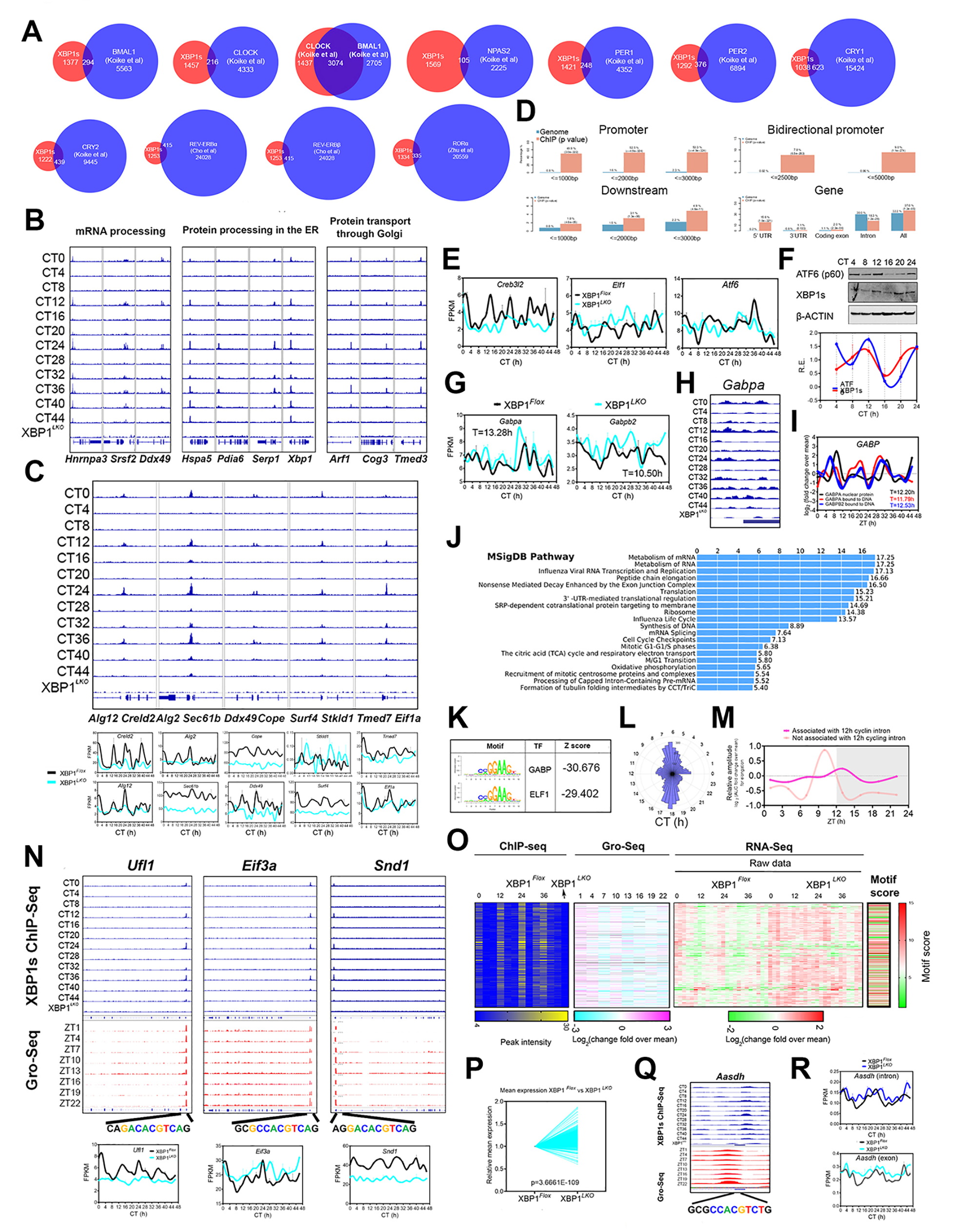

Supplement: S6 Fig — (A) Venn diagram depicting common and unique XBP1s cistrome and those of core circadian clock transcription factors compiled from [40,57,64]. (B) Snapshot of selected genes for alignment of hepatic XBP1s binding sites at different CTs in XBP1Flox and XBP1LKO mice. (C) Snapshot of selected genes for alignment of hepatic XBP1s binding sites at bidirectional promoters at different CTs in XBP1Flox and XBP1LKO mice. (D) Percentage of XBP1s cistromes identified at different positions relative to target genes compared with that of mouse genome. (E) RNA-Seq data for Creb3l2, Elf1, and Atf6 in XBP1Flox and XBP1LKO mice. (F) Western blot analysis and quantification of temporal ATF6 and XBP1s levels in mouse liver. (G) RNA-Seq data for Gabpa and Gabpb1 in XBP1Flox and XBP1LKO mice with calculated periods in XBP1Flox mice shown (panel F). (H) Snapshot of Gabpa promoter for alignment of hepatic XBP1s binding sites at different CTs in XBP1Flox and XBP1LKO mice. (I) Nuclear level of GABPA and nuclear level of GABPA and GABPB2 bound to DNA compiled from [38]. (J) GO analysis showing enriched MSiIgDB pathways and their corresponding P values for 3,730 12-h intron-mapping transcriptome without XBP1s binding sites. (K) Top enriched SeqPos motifs common to proximal promoters (1,000 bp around TSS) of 3,730 twelve-hour intron-mapping transcriptome without XBP1s binding sites. (L) Polar histogram demonstrating phase distributions of 3,730 intron-mapping 12-h cycling genes without XBP1s binding in XBP1Flox mice. (M) Log2 mean-normalized transcription elongation rates calculated from the Gro-Seq data [42] for XBP1s target genes with or without associated 12-h transcriptome. (N) Snapshot of target genes selected for alignment of hepatic XBP1s binding sites at different CTs in XBP1Flox and XBP1LKO mice as well as published Gro-Seq data [42]. Consensus XBP1s binding motifs identified at each gene promoter are also shown; (O–R) 699 genes with proximal promoter XBP1s binding but without 12-h int [file pbio.3000580.s006.tif]

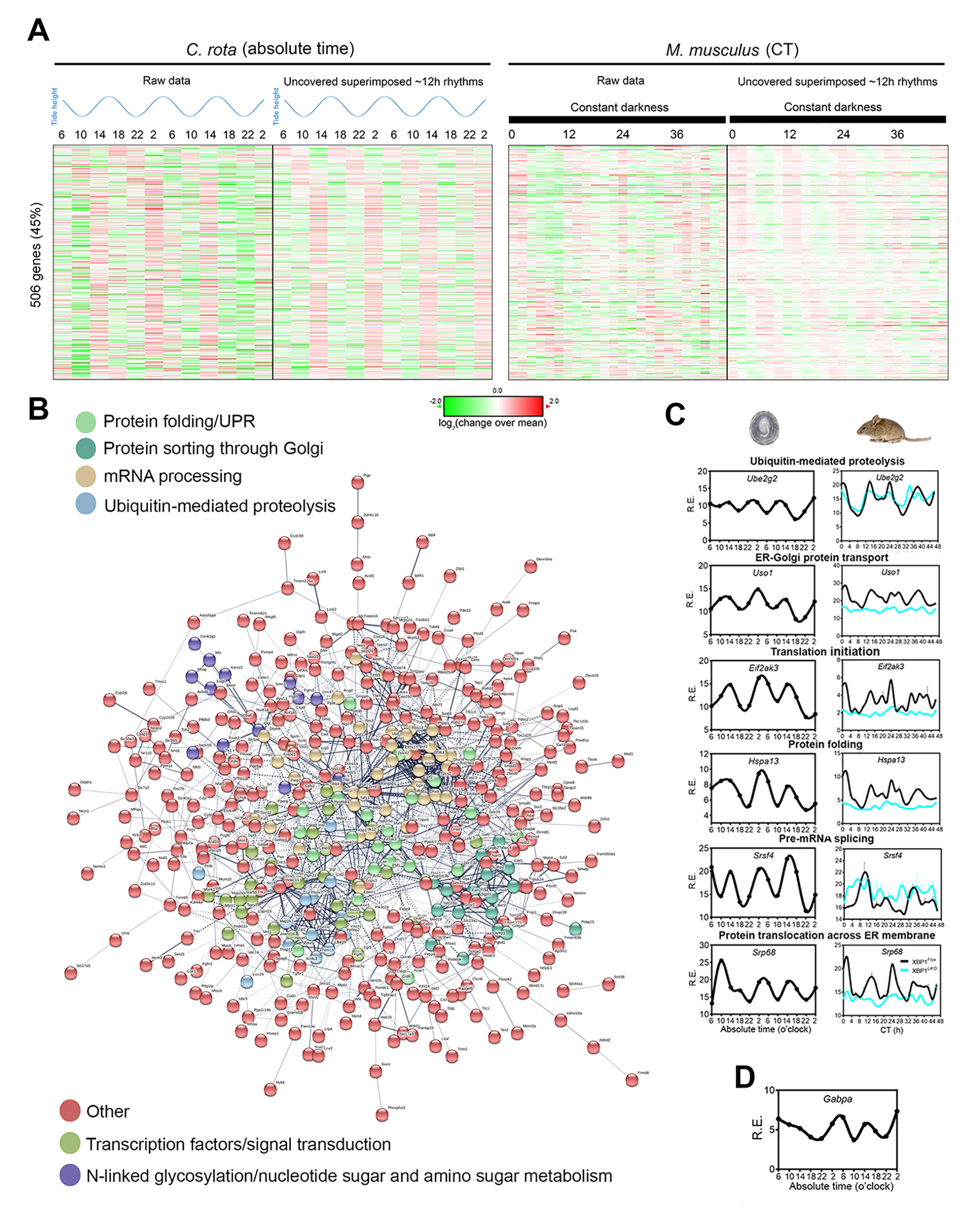

Supplement: S7 Fig — (A) Heat map of side-by-side comparison of evolutionarily conserved 12-h gene expression in C. rota [10] and mouse liver, with both raw data and superimposed circatidal rhythms shown. The level of tides corresponding to each time point is also shown. (B) Predicted interactive network construction of these conserved 12-h cycling genes using STRING [49]. Genes involved in different biological pathways are colored differently. (C) RNA-Seq data for representative genes in C. rota [10] and in XBP1Flox and XBP1LKO mice. (D) RNA-Seq data for Gabpa in C. rota. Data are graphed as the mean ± SEM (n = 2). Numerical values are available in S5 Data. A higher-resolution image in panel B is available in S3 Raw images. (TIF) [file pbio.3000580.s007.tif]

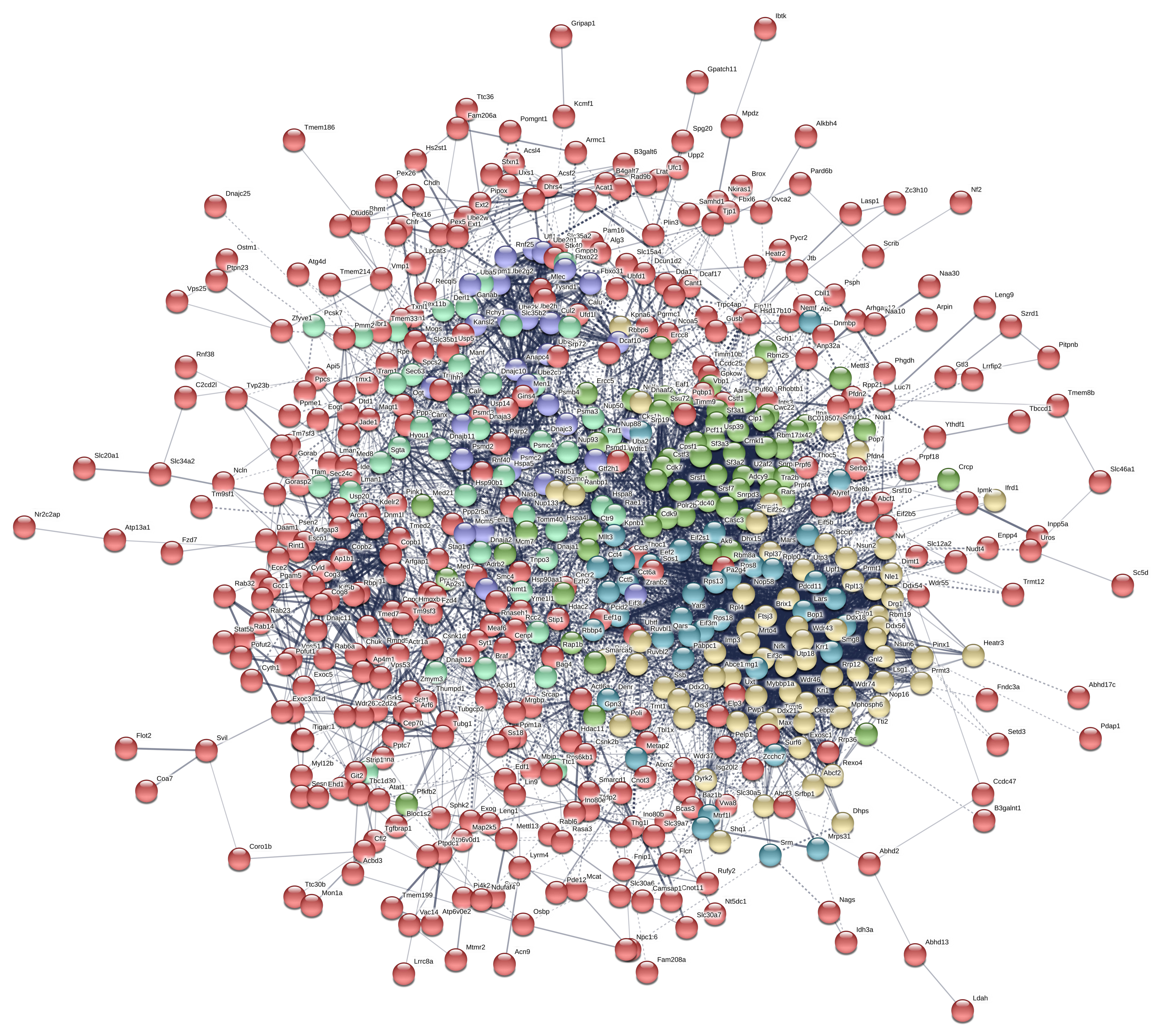

Supplement: S2 Raw image — (PDF) [file pbio.3000580.s025.pdf]

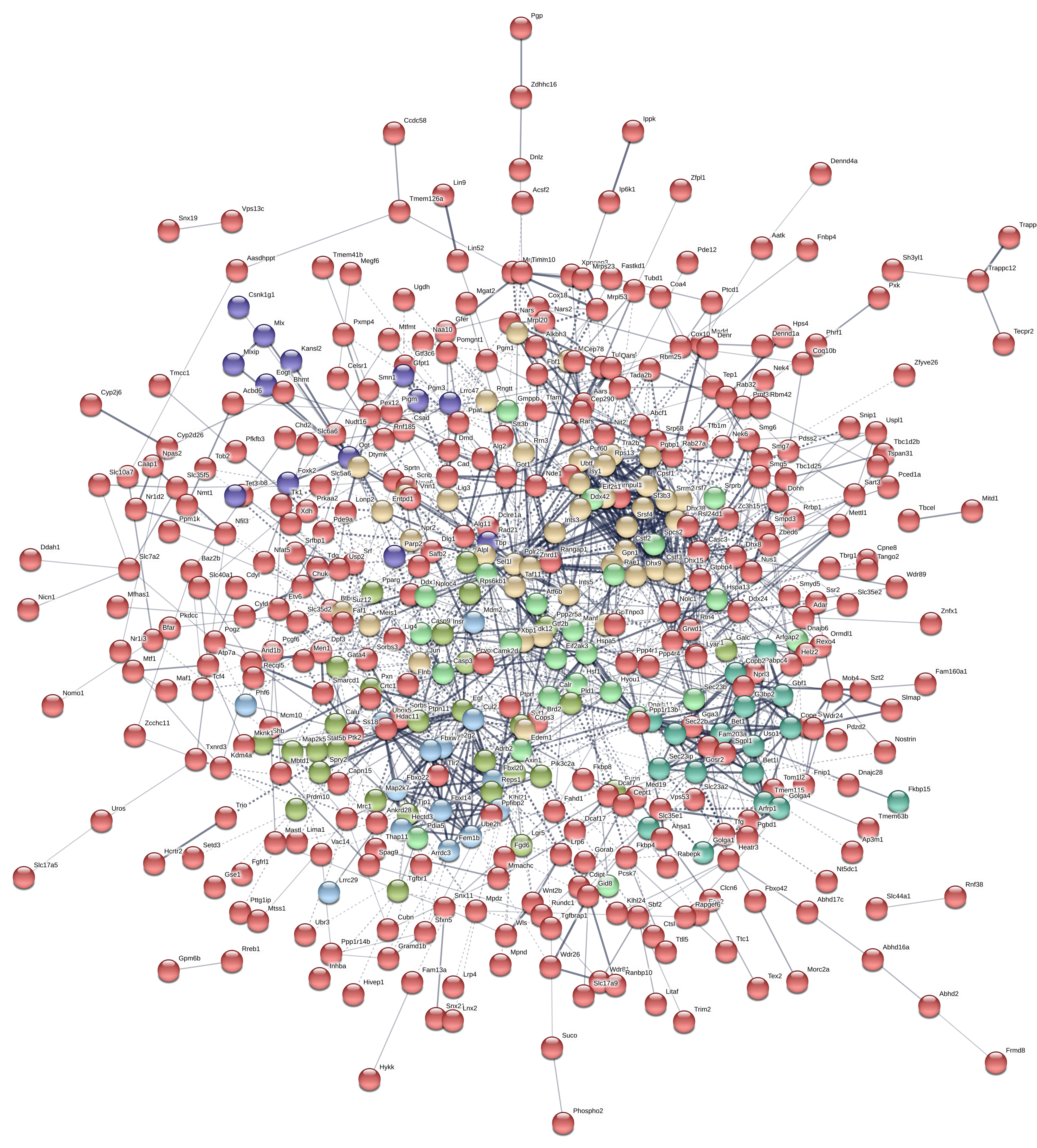

Supplement: S3 Raw image — (PDF) [file pbio.3000580.s026.pdf]
