# Supplementary material for: 12-h clock regulation of genetic information flow by XBP1s
Source: PLoS Biol. 2020 Jan 14;18(1):e3000580. doi: 10.1371/journal.pbio.3000580 (PMC6959563; doi:10.1371/journal.pbio.3000580)

S1C Fig **XBP1s blot**

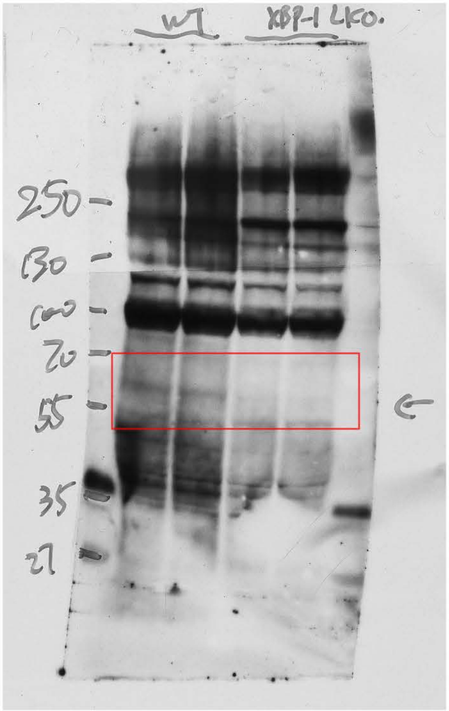

Fig 1D ACTIN BLOT

Short exposure

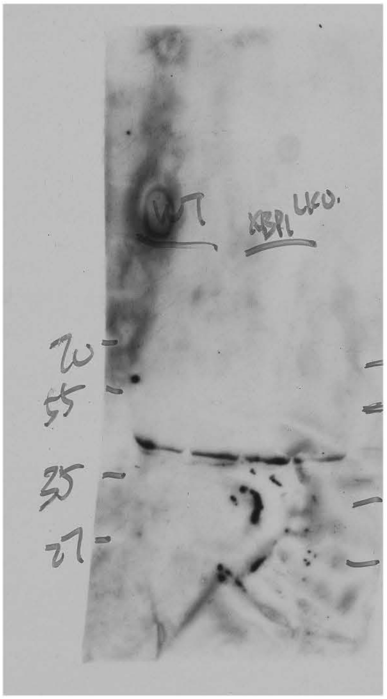

Long exposure

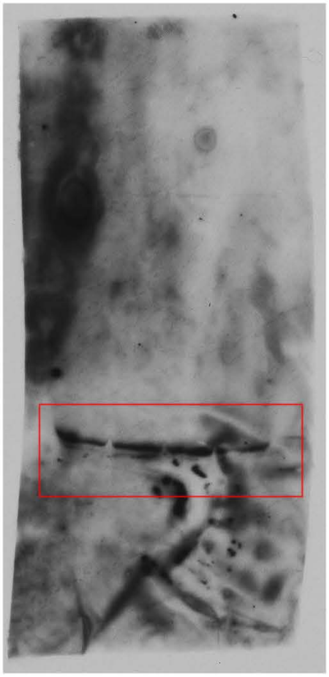

S5M Fig XBP1s

x x x x x x x x x x x x x x x x x x x x x x x x x

Scrambled siRNA
Bmal1 siRNA
Xbp1 siRNA

55kd

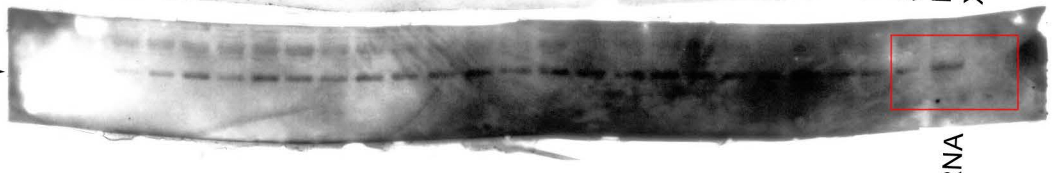

Scrambled siRNA
Bmal1 siRNA
Xbp1 siRNA

S5M Fig BMAL1

xxxxxxxxxxxxxxxxxxxxxxxxxxxxxxxxxxx

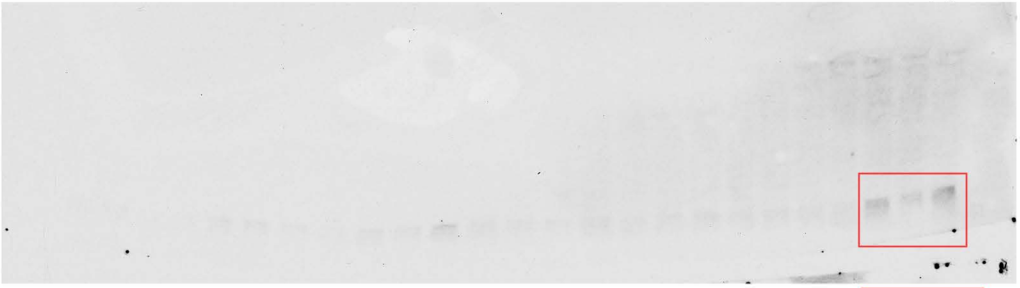

70kd

x x x x x x x x x x x x x x x x x x x x x x x x

Ponceau staining S5M Fig

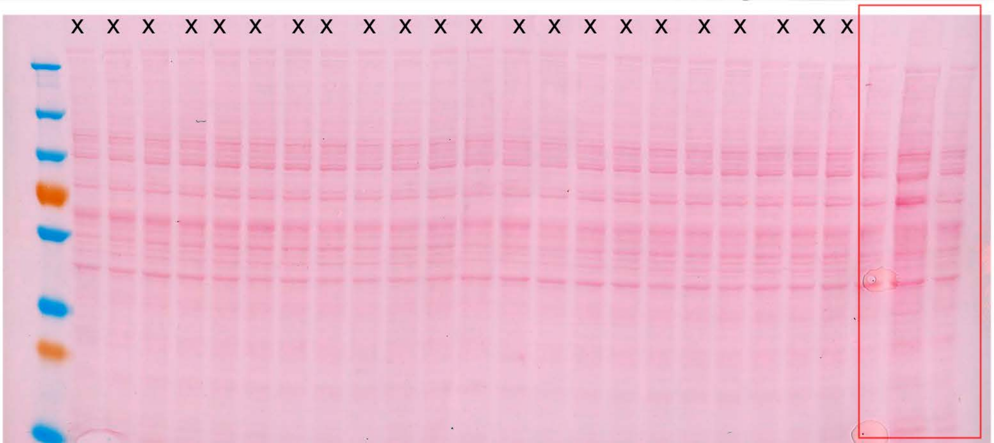

S6F Fig ATF6

CT 4 8 12 16 20 24

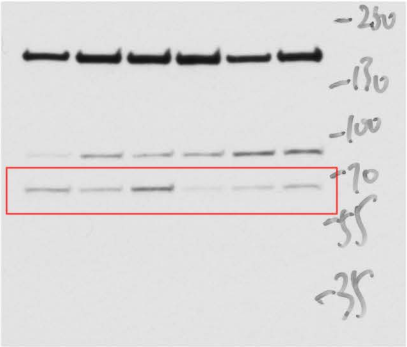

S6F Fig XBP1s

CT 4 8 12 16 20 24 X X X X X X

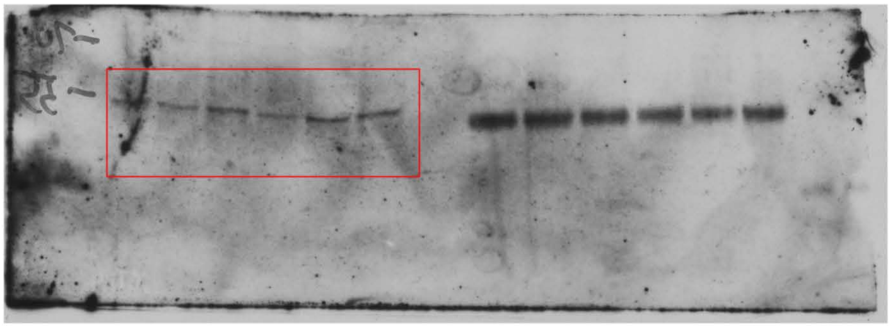

55KD

S6F Fig ACTIN

CT 4 8 12 16 20 24

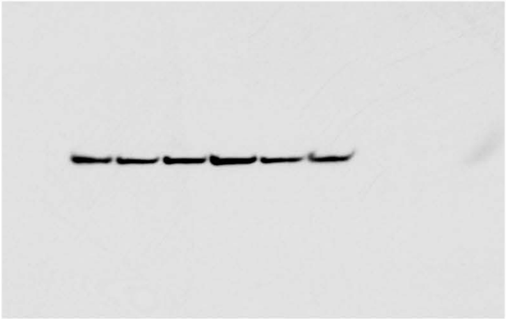

Supplement: S1 Raw images — (PDF) [file pbio.3000580.s024.pdf]
